# Sex differences in pharmacokinetics predict adverse drug reactions in women

**DOI:** 10.1186/s13293-020-00308-5

**Published:** 2020-06-05

**Authors:** Irving Zucker, Brian J. Prendergast

**Affiliations:** 1 Department of Psychology, University of California, Berkeley, 2121 Berkeley Way West, Berkeley, CA 94720 USA; 2 Department of Integrative Biology, University of California, Berkeley, 3040 VLSB, Berkeley, CA 94720 USA; 3 Department of Psychology and Committee on Neurobiology, University of Chicago, Chicago, IL 60637 USA

**Keywords:** Sex differences, Drugs, Adverse drug reactions, Pharmacokinetics

## Abstract

**Background:**

Women experience adverse drug reactions, ADRs, nearly twice as often as men, yet the role of sex as a biological factor in the generation of ADRs is poorly understood. Most drugs currently in use were approved based on clinical trials conducted on men, so women may be overmedicated. We determined whether sex differences in drug pharmacokinetics, PKs, predict sex differences in ADRs.

**Methods:**

Searches of the ISI Web of Science and PubMed databases were conducted with combinations of the terms: drugs, sex or gender, pharmacokinetics, pharmacodynamics, drug safety, drug dose, and adverse drug reaction, which yielded over 5000 articles with considerable overlap. We obtained information from each relevant article on significant sex differences in PK measures, predominantly area under the curve, peak/maximum concentrations, and clearance/elimination rates. ADRs were identified from every relevant article and recorded categorically as female-biased, male-biased, or not sex-biased.

**Results:**

For most of the FDA-approved drugs examined, elevated blood concentrations and longer elimination times were manifested by women, and these PKs were strongly linked to sex differences in ADRs. Of the 86 drugs evaluated, 76 had higher PK values in women; for 59 drugs with clinically identifiable ADRs, sex-biased PKs predicted the direction of sex-biased ADRs in 88% of cases. Ninety-six percent of drugs with female-biased PK values were associated with a higher incidence of ADRs in women than men, but only 29% of male-biased PKs predicted male-biased ADRs. Accessible PK information is available for only a small fraction of all drugs

**Conclusions:**

Sex differences in pharmacokinetics strongly predict sex-specific ADRs for women but not men. This sex difference was not explained by sex differences in body weight. The absence of sex-stratified PK information in public records for hundreds of drugs raises the concern that sex differences in PK values are widespread and of clinical significance. The common practice of prescribing equal drug doses to women and men neglects sex differences in pharmacokinetics and dimorphisms in body weight, risks overmedication of women, and contributes to female-biased adverse drug reactions. We recommend evidence-based dose reductions for women to counteract this sex bias.

## Introduction

Women historically were excluded from clinical pharmaceutical trials because of potential risks to individuals of childbearing potential. The now discredited belief that studies of men apply without modification to women also contributed to this oversight. Remediation by the US National Institute of Health (NIH) Revitalization Act of 1993 mandated enrollment of women in federally supported phase III clinical trials; a European Union initiative provided practical tools regarding sex differences in drug study design [1]. Although the inclusion of women in clinical research subsequently increased in the USA, the EU, and Australia [1–4], most studies did not provide sex-specific data analyses [5, 6]. A 2018 review of 107 NIH funded randomized control trial studies that enrolled both men and women found that only 26% reported even one outcome by sex or included both sexes as a covariate [7]; 72% simply did not include sex in their analyses. NIH policies mandated over a quarter century ago have yet to yield the intended increases in reporting by sex.

A consequence of this sex inequality hides in plain sight today: most drugs are prescribed to women and men at the same dose. Many currently prescribed drugs were approved by the US Food and Drug Administration (FDA) prior to 1993, with inadequate enrollment of female animals in preclinical research and of women in clinical trials [8]. An illustrative example is found in the sedative-hypnotic drug zolpidem, which has been marketed under several names (e.g., Ambien, Edluar, Zolpimist) since it was first approved by the FDA, 1 year before the NIH Revitalization Act. Only after decades of post-marketing reports of cognitive deficits in women given the standard male dose were sex-based dose adjustments developed (NDA 021774). Many other drugs administered in equal doses to women and men likely require re-evaluation for sex-specific dose adjustment. Even within sex, dosing is not usually weight-adjusted in adults [9]; it remains uncertain whether weight-adjusted doses will suffice to offset the majority of sex-specific ADRs. One survey of clinical pharmacology data for 300 new drug applications (NDAs) evaluated by the FDA between 1994 and 2000 [10] indicated that 31% of studies showed a possible sex effect based on pharmacokinetic (PK) sex differences greater than 20%. In the same report, 11 drugs showed a > 40% difference between males and females in PK measures, yet no dosing recommendations to consider sex were issued, implicitly based on the unsubstantiated grounds that these differences were not clinically relevant. The existing knowledge base for sex-aware prescribing lacks information on sex differences for one-third of all drugs [9, 10]. Pharmaceutical companies responsible for generating pre-approval data often fail to include information on sex differences in NDA documents, and the FDA has previously failed to enforce its own requirements before approving new drugs [11]. Consequently, potential sex differences in PK measures and their relation to unwanted side effects often remain unknown. Most of the data submitted to the FDA by drug companies are not publicly available and not subject to peer-review by the broader scientific community [9]. Regulatory agencies have historically paid insufficient attention to differences between women and men in terms of both sex and gender, which perpetuates inequalities by neglecting drug safety problems that are sex-specific. In addition, this disparity allows for misleading drug marketing [11].

The present review study inventories drugs that elicit different responses in women and men and considers sex differences in adverse drug reactions (ADRs) that occur in individuals treated with therapeutic doses of medications. The range of ADRs included, but was not limited to, nausea, headache, drowsiness, depression, excessive weight gain, cognitive deficits, seizures, hallucinations, agitation, and cardiac anomalies. Sex differentiated ADRs were operationally defined as statistically significant differences in unintended drug effects in one sex, as reported in peer-reviewed literature or in NDAs. Supplementary evidence of ADRs was obtained from *VigiBase* [12], the World Health Organization (WHO) global database of individual case safety reports (ICSRs), which since 1968 has aggregated ICSRs from member countries of the WHO Programme for International Drug Monitoring. It is important to note that pharmacovigilance datasets, including but not limited to *VigiBase*, have a number of shortcomings: the information in *VigiBase* comes from a variety of sources, and the probability that the suspected ADR is, in fact, drug-related is not the same in all cases. Thus, it cannot be proven that a specific drug caused an ADR, rather than an underlying illness or other concomitant medication. Reports submitted to *VigiBase* come from both regulated and voluntary sources, and the volume of reports about a given substance may be influenced by extent of use of the product, the nature of the ADR, and/or publicity. Lastly, many drugs are disproportionately prescribed to one sex, and *VigiBase* data do not account for the number of patients of each sex exposed to the drug [13].

In contrast, much stronger and less confounded evidence comes from clinical trials and experimentally controlled empirical studies of drugs, in which ADRs can be quantified in the context of a known number of subjects administered the drug. Because the present report is, to our knowledge, the most comprehensive attempt yet to identify sex-biased ADRs using clinically identified ADR data, it also provided an opportunity to evaluate the accuracy of *VigiBase* data in estimating sex differences in ADRs compared to estimates obtained under more controlled experimental conditions.

### Reasons why men and women respond differently to drugs

Women have a nearly 2-fold greater risk than men for exhibiting ADRs across all drug classes and are significantly more likely to be hospitalized secondary to an ADR [14–16]. This disparity is pervasive: among 668 drugs of the 20 most frequent treatment regimens in the USA, 307 (46%) report significant sex differences in ADRs [17]. In an analysis of cohort studies on 48 drugs completed between 1982 and 1997, compiling data from > 500,000 patients, Martin et al. [18] concluded that women over the age of 19 were 43 to 69% more likely to have an ADR recorded by their general practitioner. ADRs also peaked earlier in development among women (30–39 years of age) than men (50–59 years of age [18];). Women are also more likely than men to use two or more medications concurrently (polypharmacy), and women use more unique medications per year (5.0 vs. 3.7, respectively), which may contribute to increased female ADRs [19] but also renders the issue of sex-aware dosing all the more critical.

A comprehensive survey of the chemical and biological processes that underlie PKs and pharmacodynamics (PDs), and sex differences therein, is beyond the scope of the present work. We refer the reader to several thorough reviews of mechanisms relevant to PKs and PDs [20, 21] and sex differences in drug disposition [22, 23]. Here we discuss biophysical and molecular mechanisms only as required for illustration. Future analyses of drug elimination mechanisms (e.g., via CYP3A4) may facilitate extrapolation of the present results to a larger sample of drugs.

In general, drug disposition occurs through separate phases: absorption, distribution, bioavailability, metabolism, and excretion, and sex differences have been documented for each phase [22, 23]. Women generally have a lower body weight and organ size and a higher percentage of body fat, which affects the absorption and distribution of drugs. The larger the volume of distribution (V_d_), the more likely the drug will be found in body tissues.

A number of biological, psychological, and cultural factors may contribute to why sex is such a strong risk factors for ADRs, including the following: sex differences in PKs and PDs, sex-specific organizational (early life) and activational (peripubertal through adulthood) endogenous steroid hormone exposure, and sex differences in exogenously administered steroids, higher rates of polypharmacy in women, sex differences in the expression of somatoform disorders, and sex differences in reporting rates [24].

Drug clearance is strongly linked to sex-specific expression of metabolic enzyme systems [25–27]; renal clearance of drugs is decreased in women because of a relatively lower glomerular filtration rate compared to men [28]. Women have a slower gastric emptying time and lower gastric pH, lower plasma volume, body mass index, average organ blood flow, and total body water differences, all of which affect drug distribution and PKs [29]. Responses to drugs are also affected by physiological changes during the menstrual cycle. The striking hormonal variations across days over the course of the human menstrual have no parallel in men in which hormonal variations largely occur within rather than across days [30].

The anticoagulant drug lepirudin is excreted by the kidney with systemic clearance in women about 25% lower than in men (NDA 020807 [31]). But PK variables do not translate linearly into phenotypes; thus, in women lepirudin is detectible in the circulation for up to 48 h, compared to just 2 h in men, which greatly increases the potential for undesired bleeding [32]; indeed, in this example, low molecular weight heparin-induced thrombocytopenia is a clinically important ADR which occurs more frequently in women than men [33], a difference that corresponded to much higher drug exposure as indicated by female-bias in multiple PK measures.

Arpon et al. [34] maintain that dosing adjusted according to a range of patient-specific factors is of large and increasing clinical and financial concern but lament that the amount of body weight adjusted dosing change that occurs in clinical practice is presently unknown. Adjustments for body mass in most cases do not ameliorate the high incidence of female ADRs. Thus, a multivariate regression analysis controlling for age, body mass index, and number of prescribed drugs identified a strong and significant effect of female sex on the increased risk of encountering ADRs, indicating that the sex disparity in ADRs does not merely reflect body mass masquerading as an effect of sex [35].

Most drugs are not administered on a milligram/kilogram basis but as a “one size fits all” dose, leading to higher exposures in women [10]. Under optimal circumstances, the dose should be based on milligram/kilogram body weight, or titrated to the desired clinical effect [36]. Correction for height, weight, surface area, or body composition eliminates a minority of sex-dependent PK differences [10]. The inference that weight-corrected PKs are comparable between men and women is not generally warranted but must be examined on a case-by-case basis, if data on both sexes exist. If for a given drug with a sexually differentiated pattern of ADR expression, correction for body weight eliminates the sex differences in PKs, this may or may not have bearing on PK-driven exposure leading to sex-differentiated ADRs. In short, sex differences in PK may be sufficient but not necessary for the manifestation of sex differences in ADRs.

PKs differ in men and women for many drugs [4, 21–23, 37–40]; this impacts drug efficacy and toxicity [41, 42]. Data from bioequivalence trials identified significant sex differences in PKs in ~ 28% of data sets [43]. Despite these differences, sex-specific dosing recommendations are absent for most drugs [37]. When women consistently experience less therapeutic effect or more adverse effects, a change in dosing regimen may be necessary.

Sex-related differences in PKs present a more significant challenge for medications with a low therapeutic index, i.e., those in which the lowest effective dose approaches toxic concentrations [44, 45]. If the therapeutic index is narrow, these differences are more likely to become clinically significant. A detailed study of sex differences for drugs with either steep dose response curves or narrow therapeutic indices is warranted [46]. For drugs with a relatively wide therapeutic index, a fixed dosing regimen is less of an impediment, but even in these instances, selecting the lowest effective dose would be prudent in women, at a minimum to decrease the potential for ADRs, as women are more likely than men to be prescribed more than one drug at a time.

Population PK studies often rely on sparse blood sampling collected from many subjects in phase II and III clinical trials. PK information is included in only a small minority of approved drug labels [10]; among > 2500 compounds listed in the Physicians Desk Reference (PDR), only 88 (fewer than 4%) presented population PK information in labelling [47]. PK sex difference data routinely are derived from small clinical pharmacology studies, typically enrolling 12–24 healthy subjects. Studies with such relatively small sample sizes have lower statistical power: they may be adequate to detect only very large sex differences in PK attributes, but as effect sizes decrease, these under-powered experimental designs generate widespread type II statistical errors—they become less and less capable of identifying real sex differences.

### Aims

Here we examine relations between sex differences in drug PKs and ADRs to critically evaluate the hypothesis that drug exposure (PKs) and bodily responses to drugs (PDs; more specifically, clinically unintended effects, or ADRs) should be considered in the development of rational, feasible sex-based dosing adjustments. We conclude that such considerations are presently missing and recommend that sex-based dosing recommendations be disseminated to physicians and appear on drug labels. In many cases, these changes can be implemented at little cost. As demonstrated below, the data required to implement these procedures already exist for a number of drugs but have been ignored.

To determine the extent to which sex as a biological variable has been incorporated in the development of therapeutic pharmaceuticals, we reviewed whether sex differences in PK data were identified for any given drug and, where present, if such differences were incorporated into recommendations for sex-aware dosing/prescribing. Additionally, we reviewed an extensive literature on sex-specific ADRs, to evaluate whether sex differences in PK data predict sex disparities in ADRs. The hypothesis tested was that sex differences in PKs, specifically higher drug exposure in women than men, would be associated with clinically significant sex differences in ADRs. Support for this hypothesis, based on the broad net cast in the present investigation, would support dose reductions in women for the drugs under investigation here, and perhaps warrant extrapolation to any drugs for which sex differences in PKs exist. Several recent comprehensive reviews have addressed sex differences in drug treatment targeting specific diseases [48–53]. Here we report, for the first time, substantial sex differences in PKs and ADRs for 86 drugs, spanning multiple (> 10) therapeutic categories that support dose adjustments for women.

## Methods

### Objective

To examine whether sex differences in PKs are related to sex differences in ADRs, we reviewed empirical studies that measured PKs in females and males treated with FDA-approved drugs and quantitative reports documenting ADRs in both sexes.

### Search strategy, analysis steps, and criteria for study inclusion

Multiple searches of the ISI Web of Science database (core collection, basic search; Thompson Scientific) were conducted throughout 2018–19 with the terms: *drugs* and *sex differences* and *pharmacokinetics* (848 articles), *drugs* and *gender* and *pharmacokinetics* (1548 articles), *drugs* and *sex differences* and *pharmacodynamics* (385 articles), *drug safety* and *dose* and *sex* (651 article), *drug safety* and *dose* and *gender* (462 articles), *adverse drug reactions* and *sex* (912 articles), *adverse drug reaction* and *gender* (687 articles), which yielded 5493 articles, with considerable overlap. All years were included in the Web of Science and Pub Med searches. Only English language articles were accessed. For a publication to be included in the survey, it had to contain data on sex differences in pharmacokinetics and/or adverse drug responses. The Thompson Reuters Web of Science and the NCBI PubMed databases were searched with no date restrictions; the last search was completed in January 2020. The WHO’s VigiBase database was searched with no date or geographic restrictions.

### Data extraction

#### PK measures

Each article was examined for relevance with additional searches conducted in PubMed and Web of Science for specific drugs; relevant publications identified in target articles were also inspected. Many drugs do not have a sex bias in pharmacokinetics. In an unknown number of instances, this reflects a true absence of sex differences based on empirical findings. For many drugs, however, the available PK data are lacking because female subjects were either not included in study design, or data were not reported by sex. Only studies of drugs that have a sex bias in pharmacokinetics were considered. Risk bias was examined in all articles to determine whether both sexes were included, whether sample size was adequate, and if subjects enrolled were adults or children. Sample size was considered adequate if there were 10–12 subjects/group. Several articles reporting statistically significant results with smaller sample sizes were reported but flagged with a disclaimer. From each relevant article, data on sex differences in PK measures was extracted; the majority of these measures were in units of either area under the curve [AUC] or peak/maximum concentration [C_max_]; less frequently, data were available for drug half-life [*t*_1/2_], clearance/elimination measures conducted over fixed time intervals, systemic exposure, V_d_, and T_max_.

#### ADR measures

ADRs were identified from every relevant article and recorded categorically as female-biased or male-biased. The EU database of ADR reports and the WHO’s ADR monitoring website, also known as *VigiBase*, were searched. ADR data from this site are listed in instances where we were unable to find primary reports of ADRs for a specific drug. The ratio of women:men reporting ADRs was computed, and a value that deviated by more than 20% from 1.0 in either direction indicated that *VigiBase* reports were sex biased: values > 1.2 and < 0.80 indicated female and male sex biases, respectively. Values between 0.80–1.20 (inclusive) were interpreted as indicating no sex bias in ADRs. As discussed above, a major limitation of *VigiBase* is that it does not account for differences in the frequency with which women and men are treated with a specific drug. To directly examine this limitation in the context of sex differences in ADRs, we separately compared the prevalence of clinical ADRs (from data in the literature) with sex-specific reporting rates in *VigiBase*.

Label information and PK and PD data, where available, were obtained from the *Drugs@FDA* database [54]. Only data from human subjects were considered. Recreational drugs including alcohol, cocaine, nicotine, heroin, most psychedelics, and drugs involved in treatment of AIDS were beyond the scope of this review. For each drug, we report information published in peer-reviewed journals, from FDA NDA forms, and approved product labels. PK data are presented first, followed by ADRs.

Generally, sex differences refer to differences between women and men in the gonads, genitals, hormones, chromosome, and gene complements. Gender differences are more difficult to define and usually encompass gender roles and gender identity. With a few exceptions, we use the term sex differences to describe differences in pharmacokinetics, pharmacodynamics, and adverse drug reactions.

### Data reduction

Results are provided in the form of a descriptive analyses of whether studies with sex-specific patterns of PK bias predict sex-specific patterns of ADR bias. Only studies with clear sex differences in pharmacokinetics were included in the data synthesis. Chi-square analyses also evaluated whether PK biases predicted VigiBase data.

Among the 86 drugs that were identified as having sex-specific PK data, one compound, tirilazad (ATC code N07XX01), exhibited clear sex-biased PK values (greater exposure in men), but neither clinical ADRs nor sufficient entries in *VigiBase* existed; as a result, tirilizad is discussed below but not included in final tabular calculations. Another drug, theophylline (R03DA04), exhibited conflicting PK data (i.e., some PK measures indicated greater exposure in women whereas others indicated greater exposure in men), no experimentally identified ADRs, but clear sex-biased adverse responses in post marketing reports and is therefore included in both the discussion and tabular calculations below. Lastly, for 4 drugs (mirabegron, sertraline, aspirin, and clozapine), ADRs were not uniformly present in one sex. For mirabegron, a number of publications indicated a female bias in ADRs, and for aspirin and sertraline, the overall number of ADRs reported was greater for women; these drugs were classified as having female-biased ADRs. For clozapine, empirical reports and meta-analyses identified over a dozen sexually differentiated ADRs, but overall, more ADRs occurred in men than in women, so clozapine was classified as having a male-biased ADR profile.

## Results

Data for all drugs are contained in Additional file 1 which lists sex-specific PKs for drugs from every individual report. Full information is included in this file narrative, along with measures of central tendency (mean, median) and variance (SD, SEM, range) from the original reports where available. These results are summarized in Tables 1, 2, and 3, which list the specific PK measures for each drug, their direction of sex bias, whether ADRs were female- or male-biased, and whether PK findings and ADR data were sex-concordant or sex-discordant.

**Table 1:** Summary of sex-biased PK measures and concordance with clinical ADRs

WHO ATC category	Drug^a^	PK measure^b^	Female-biased ADRs	Male-biased ADRs	PK vs ADR
Alimentary tract, metabolism	Liraglutide	CL_T_	headache, vomiting, nausea	--	Concordant
	**Ranitidine**	***AUC*, T**_**max**_	--	**Duodenal damage**	Concordant
	Rosiglitazone	CL_T_	Fracture	--	Concordant
Blood and blood forming organs	Heparin	CL_T_	blood disorders, lymphatic disorders, bleeding (60 yoa)	--	Concordant
	Aspirin	*AUC*, Cl_T_, t_1/2_	elevated fibrinogen, CVD in T2D	**Bleeding**	Concordant
	Warfarin	CL_T_	Bleeding	--	Concordant
	Clopidogrel	*AUC*, C_max_	fracture, bleeding, GI symptoms, IBD	--	Concordant
	Dabigatran	*AUC*	Bleeding	--	Concordant
Cardiovascular system	Torasemide	*AUC*, C_max_, Cl_T_,t_1/2_	Hospitalization	--	Concordant
	Pravastatin	AUC, C_max_	CHD	--	Concordant
	**Amlodipine**	**CL**_**T**_	Edema, flushing, palpitations	--	Discordant
	Digoxin	CL_T_	Mortality	--	Concordant
	Verapamil	CL_T_	Constipation, edema, fatigue, headache	--	Concordant
	Aliskiren	*AUC*, C_max_	Diarrhea	--	Concordant
	Losartan	*AUC*, C_max_	--	**Angina, mortality**	Discordant
	Propranolol	*AUC,* C_max_, Cl_T_	dizziness muscle pain headache dry mouth	--	Concordant
	Dofetilide	SysExp	TdP	--	Concordant
Genito-urinary system, sex hormones	Mirabegron	*AUC*, C_max_, SysExp	Treatment discontinuation (CAN), treatment discontinuation (CZE)	**Treatment discontinuation (UK)**	Concordant
	Darifenacin	*AUC*, C_max_, Cl_T_	F > M, ADRs not specified	--	Concordant
	Trospium	*AUC*, C_max_	Cognitive impairments	--	Concordant
Systemic hormonal preparations	Prednisone	*AUC,* Cl_T_	Depression, fatigue, hair loss, mood swings, weight gain	--	Concordant
Anti-infectives	Levofloxacin	Cl_T_, SysExp	Fluoroquinolone toxicity	--	Concordant
	**Erythromycin**	**CL**_**T**_	TdP	--	Discordant
	Voriconazole	*AUC,* C_max_	cardiac QTc symptoms	--	Concordant
Antineoplastics, immunomodulators	**Cyclosporin**	**CL**_**T**_	--	--	Discordant*
	Fluorouracil	*AUC*, Cl_T_	Stomatitis, leukopenia, alopecia, diarrhea, mucositis	--	Concordant
	Paclitaxel	CL_T_	Myocardial infarction, death, lesion revascularization	--	Concordant
	Capecitabine	*AUC*	Dose-limiting toxicity	--	Concordant
	Infliximab	CL_T_	Allergic reactions	--	Concordant
	Adalimumab	CL_T_	Allergic reactions	--	Concordant
Anesthetics, analgesics	Morphine	CL_T_	Respiratory depression, hypoxic sensitivity, emesis, nausea	--	Concordant
	Oxycodone	CL_T_	Nausea, pruritus, negative affect	--	Concordant
	Buprenorphine	*AUC,* C_max_	Sleep disturbance	--	Concordant
	Tramadol	*AUC,* C_max_	Treatment discontinuation	--	Concordant
	Zolmitriptan	*AUC,* C_max_	Face and neck presure	--	Concordant
	**Ketamine**	**Cl**_**T**_	--	**Verbal learning, subjective memory**	Concordant
Anti-epileptics, anti-Parkinson’s	**Carbamazepine**	**Cl**_**T**_**, t**_**1/2**_	Cognitive impairment, elevated LDL/HDL	--	Discordant
	Gabapentin	C_max_	Dizziness, somnolescence, nausea	--	Concordant
	Perampanel	*AUC*, Cl_T_	Dizziness, headache, treatment discontinuation	--	Concordant
	Pramipexole	*AUC*, C_max_	Nausea, fatigue	--	Concordant
Psycholeptics	Olanzapine	CL_T_	Severe weight gain		Concordant
	Clozapine	CL_T_	Blood glucose, laxative use, obesity, type II diabetes, neutropenia, leukopenia, weight gain	**Hypertension, increases in BMI, elevated homocysteine, increased basal metabolic rate, increased plasma lipids, QTc symptoms, blood dyscrasias, myocarditis, cardiomyopathy**	Discordant
	Risperidone	CL_T_	Hyperprolactinemia, neurological effects, headache, hypotension	--	Concordant
	Aripiprazol	*AUC*, C_max_	Blood pressure, heart rate, elongated QTc	--	Concordant
	Diazepam	t_1/2_	Psychomotor impairment	--	Concordant
	Zolpidem	*AUC*, C_max_, Cl_T_	Cognitive impairment, driving impairment	--	Concordant
	Eszopiclone	*AUC*	Dysgeusia	--	Concordant
Psychoanaleptics	Imipramine	CL_T_	Dry mouth, constipation, sweating, tremor, treatment discontinuation	--	Concordant
	Nortriptyline	CL_T_	Dry mouth	--	Concordant
	Fluoxetine	CL_T_	Hypercortisolemia, elevated albumin, elevated tryptophan, suicidal ideation	--	Concordant
	Citalopram	CL_T_	Elevated ADH	--	Concordant
	Sertraline	*AUC*, Cl_T_, t_1/2_	Cholesterol, nausea, dizziness, delusions	**Dyspepsia, sexual dysfunction, urinary frequency**	Concordant
	Bupropion	*AUC*, C_max_, t_1/2_	EEG abnormalities, seizure	--	Concordant
	**Methylphenidate**	***AUC***	Anxiety disorder	--	Discordant
Antiparasitics	Primaquine	*AUC*, C_max_	Nausea	--	Concordant
Respiratory	Terfenadine	C_max_	TdP	--	Concordant
	Fexofenadine	*AUC*, C_max_	Visual attention deficits, reaction time deficits, driving impairments	--	Concordant
Miscellaneous	MDMA	CL_T_	Elevated copeptin, hyponatremia	--	Concordant
	Cannabis	CL_T_	Increased subjective emotional responses, problematic useintrovertive anhedonia	--	Concordant

PK abbreviations: *AUC* area under the curve, *C*_*max*_ maximum circulating concentration*, T*_*max*_ time required to reach C_max_ value, *t*_*1/2*_ elimination half-life, *Cl*_*T*_ clearance time, exposure, or plasma concentration after a fixed time interval, *SysExp* systemic exposure

*No evidence of sex differences in ADRs

^a^Font style of drug name indicates direction of PK drug exposure bias: (normal font: F > M; **bold font**: M > F)

^b^Font style of PK measure indicates direction of greater drug exposure (normal: F > M; **bold**: M > F)

**Table 2:** Concordance between sex-biased PKs and VigiBase sex ratios

WHO ATC category	Drug name/PK bias^a^	PK measure^b^	VB ratio^c^	Concordance
Alimentary tract, metabolism	Ondansetron	*AUC*, C_max_	2.14	Concordant
Blood and blood forming organs	Icatibant	*AUC*, C_max_	3.18	Concordant
Cardiovascular system	Metoprolol	*AUC*, C_max_	1.26	Concordant
	Labetalol	C_max_, Cl_T_	1.50	Concordant
	**Nifedipine**	**Cl**_**T**_	1.29	Discordant^1^
	Pitavastatin	*AUC*, C_max_	1.12	Discordant^2^
Systemic hormonal preparations	**Methylprednisolone**	***AUC*, t**_**1/2**_**, Cl**_**T**_	1.38	Discordant^1^
Musculoskeletal	**Ibuprofen**	***AUC,*C**_**max**_**, t**_**1/2**_**, Cl**_**T**_	1.46	Discordant^1^
Anesthetics, analgesics	Lignocaine	Cl_T_	1.64	Condordant
	Propofol	*AUC,***Cl**_**T**_	1.45	Concordant
	Butorphanol	*AUC,* C_max_	3.23	Concordant
	Paracetamol	Cl_T_	1.68	Concordant
Anti-epileptics	Ezogabine	*AUC,* C_max_, *Cl*_*T*_	0.90	Discordant^2^
Psycholeptics	Clobazam	t_1/2_	1.11	Discordant^2^
	Sertindole	*AUC*, C_max_	1.06	Discordant^2^
	Thiothixene	Cl_T_	1.35	Concordant
	Oxazepam	Cl_T_	1.31	Concordant
	Temazepam	t_1/2_, Cl_T_	1.34	Concordant
	Zopiclone	*AUC*, C_max_	1.55	Concordant
Psychoanaleptics	Memantine	SysExp	1.50	Condordant
	Desipramine	*AUC*, Cl_T_	1.59	Concordant
	Clomipramine	Cl_T_	1.57	Concordant
	Paroxetine	Cl_T_	2.00	Concordant
	Fluvoxamine	Cl_T_	1.73	Concordant
	Mirtazapine	Cl_T_	1.53	Concordant
Respiratory	Cetirizine	Cl_T_	1.76	Concordant

PK abbreviations: *AUC* area under the curve, *Cmax* maximum circulating concentration, *Tmax* time required to reach Cmax value, *t1/2* elimination half-life, *ClT* clearance time, exposure, or plasma concentration after a fixed time interval, *SysExp* systemic exposure, *DurAbs* duration of absorption

^a^Font style of drug name indicates direction of PK drug exposure bias: (normal: F > M; **bold**: M > F)

^b^Font style of PK measure indicates direction of greater PK drug exposure: (normal: F > M; **bold**: M > F; *italic*: inconsistent)

^c^F-to-M reporting ratio of suspected ADRs. Threshold for designation of sex bias = ± 20%

^1^False positive

^2^False negative

**Table 3:** Sex ratios of adverse drug event reports in *VigiBase* among 59 drugs with sex-biased ADRs

WHO ATC category	Drug name/PK bias^**a**^	ADR bias	VB ratio^**b**^	ADR-VB relation
Alimentary tract, metabolism	Liraglutide	Females	1.79	Concordant
	**Ranitidine**	Males	1.52	Discordant^1^
	Rosiglitazone	Females	0.79	Discordant^1^
Blood and blood forming organ	Heparin	Females	0.92	Discordant^2^
	Aspirin	Females	0.85	Discordant^2^
	Warfarin	Females	0.91	Discordant^2^
	Clopidogrel	Females	0.69	Discordant^2^
	Dabigatran	Females	0.93	Discordant^2^
Cardiovascular system	Torasemide	Females	1.15	Discordant^2^
	Pravastatin	Females	1.13	Discordant^2^
	**Amlodipine**	Females	1.41	Concordant
	Digoxin	Females	1.26	Concordant
	Verapimil	Females	1.56	Concordant
	Aliskiren	Females	1.27	Concordant
	Losartan	Males	1.41	Discordant^1^
	Propranolol	Females	1.72	Concordant
	Dofetilide	Females	0.92	Discordant^2^
Genito-urinary system, sex hormones	Mirabegron	Females	1.95	Concordant
	Darifenacin	Females	2.48	Concordant
	Trospium	Females	2.14	Concordant
Systemic hormonal preparations	Prednisone	Females	1.17	Discordant^2^
Anti-infectives	Levofloxacin	Females	1.26	Concordant
	**Erythromycin**	Females	1.70	Concordant
	Voriconazole	Females	0.65	Discordant^1^
Antineoplastics, immunomodulators	**Cyclosporin**	Neither	1.25	Discordant^1^
	Fluorouracil	Females	1.00	Discordant^2^
	Paclitaxel	Females	2.66	Concordant
	Capecitabine	Females	1.77	Concordant
	Infliximab	Females	1.51	Concordant
	Adalimumab	Females	2.02	Concordant
Anesthetics, analgesics	Morphine	Females	1.57	Concordant
	Oxycodone	Females	1.01	Discordant^2^
	Buprenorphine	Females	1.61	Concordant
	Tramadol	Females	1.71	Concordant
	Zolmitriptan	Females	5.14	Concordant
	**Ketamine**	Males	1.07	Discordant^2^
Anti-epileptics, anti-Parkinson’s	**Carbamazepine**	Females	1.26	Concordant
	Gabapentin	Females	1.84	Concordant
	Perampanel	Females	1.18	Discordant^2^
	Pramipexole	females	1.19	Discordant^2^
Psycholeptics	Olanzapine	Females	0.92	Discordant^2^
	Clozapine	Males	0.61	Concordant
	Risperidone	Females	0.53	Discordant^1^
	Aripiprazol	Females	1.27	Concordant
	Diazepam	Females	1.17	Discordant^2^
	Zolpidem	Females	1.48	Concordant
	Eszopiclone	Females	1.85	Concordant
Psychoanaleptics	Imipramine	Females	1.61	Concordant
	Nortriptyline	Females	2.10	Concordant
	Fluoxetine	Females	2.29	Concordant
	Citalopram	Females	1.95	Concordant
	Sertraline	Females	2.00	Concordant
	Bupropion	Females	1.79	Concordant
	**Methylphenidate**	Females	0.47	Discordant^1^
Antiparasitics	Primaquine	Females	0.55	Discordant^1^
Respiratory	Terfenadine	Females	1.66	Concordant
	Fexofenadine	Females	2.05	Concordant
Miscellaneous	MDMA	females	0.43	Discordant^1^
	Cannabis	females	0.51	Discordant^1^

Threshold for designation of a *VigiBase* reporting sex bias was set at ± 20%

^a^Font style of drug name indicates direction of PK drug exposure bias: (normal: F > M; **bold**: M > F)

^b^Female-to-male (F:M) ratio of suspected ADRs

^1^“false sex” error

^2^“false null” error

We identified 86 drugs with significant sex differences in PKs, based on C_max_ and AUC values or drug distribution and elimination rates. Many drugs with striking sex differences in ADRs did not make it onto this list because there were no available PK data, almost always because drugs were approved prior to the year 2000, and thus the relevant information is not available in the online FDA database.

The correspondence of sex differences in PKs with sex differences in ADRs was striking. In 88% of instances, a sex difference in PKs was linked to a similar sex difference in ADRs (52 of 59 drugs were PK-ADR “concordant,” summarized in Table 1), a substantially higher incidence than the 46% sex difference in ADRs across all drugs assessed without regard to PK differences [17]; thus, including PK data in the consideration of ADRs, and stratifying analyses of drugs both by the presence, and by the direction, of PK differences greatly clarifies patterns of sex differences in ADRs. Overall, 76 of 86 drugs (88%) had higher PK values in women than men (Tables 1 and 2), and among the 59 drugs for which ADR data were available. Of course, it remains possible that sex differences in PK parallel sex differences in ADRs independently. Ninety-six percent with higher PK values in women also induced a higher incidence of ADRs in women. For 7 drugs, male PK values exceeded those of females, but this sex difference positively predicted male-biased ADRs in only 2 of 7 instances (29%; 4 exhibited female-biased ADRs and 1 no bias; Table 2). In the absence of any obvious reason to suspect that our sample is not representative of the more than thousands of drugs approved for human use by the FDA (US-FDA, 2019), the present data suggest that elevated drug concentrations and decreased elimination times are far more prevalent in women than men and present a quantifiable and major health risk for women.

### Comparison of VigiBase adverse event reporting with empirical clinical data

For 27 of the 86 drugs surveyed, *VigiBase* was the only source available for data on sex differences in ADRs (Table 2). Similar to the drugs summarized in Table 1, 24 of these 27 drugs (89%) exhibited female-biased PK values (Table 1, 52/59; Table 2, 24/27; χ^2^ = 0.01, *P* > 0.9), among these drugs, sex-biased PKs predicted sex-biased VigiBase ADR reporting ratios (hereafter, “VB ratios”) in 74% of instances (20 of 27 drugs were PK–VB ratio “concordant,” Table 2). To examine how closely estimates of sex differences in ADRs inferred from VB ratios corresponded to actual sex differences in ADRs gathered from the empirical clinical literature, we calculated VB ratios for the 59 compounds in Table 1, i.e., drugs for which actual ADR information was available. This analysis indicates that sex-biased patterns of case reports in the *VigiBase* pharmacovigilance database correctly predict actual sex differences in ADRs in only 33 of 59 cases (56% of drugs were ADR–VB ratio “concordant,” Table 3); concordance between ADR bias and VB ratios did not differ between drugs that had male- vs. female-biased PK values: 3 of 7 drugs (43%) with male-biased PKs were ADR–VB ratio concordant, 30 of 52 drugs (58%) with female-biased PKs were ADR–VB ratio concordant; χ^2^ = 0.55, *P* > 0.4. *VigiBase* indicated no sex bias in adverse event reporting (VB ratios between 0.80 and 1.20, as defined above, a “false null” error) for 15 of 26 (58%) ADR–VB ratio discordant drugs, and a sex-biased VB ratio in a direction opposite to that of the actual ADR bias (e.g., a male-biased VB ratio, when real ADRs are female biased, a “false sex” error) in 11 of 26 (42%) discordant drugs (Table 3). Pharmacovigilance databases are useful as broad indicators, but the present analysis underscores the limitations inherent in *VigiBase* ADRs.

## Discussion

### PK values predict ADRs but only in women

The present results reveal a striking sex difference in pharmacokinetics: among patients administered a standard drug dose, females are exposed to higher blood drug concentrations and longer drug elimination times than males. This likely contributes to the near doubling of adverse drug reactions in female patients, raising the possibility that women are routinely overmedicated. Whether this relation also holds for the thousands of drugs for which sex-stratified PK data are not publicly available remains to be investigated, but an affirmative answer would be consistent with our analysis. The present results also support the argument that for drugs with a sufficiently broad therapeutic index, dose reductions in women would yield a reduction in side effects.

A number of reports have identified links between sex-specific patterns of PK values, PDs, and ADRs [48, 51, 55], but to our knowledge, the present analysis is the first to document that sex differences in PKs positively predict sex differences in ADRs broadly across multiple categories of FDA-approved pharmaceuticals. These data indicate that if a significant, female-biased PK sex difference is identified, then there is an overwhelmingly high probability that a clinically identifiable female-biased ADR will co-occur. Although it remains to be definitively established that women routinely are overmedicated, the present data are consistent with this conjecture for the drugs reviewed here, as well as for the thousands of drugs administered in equivalent doses to women and men. The intended efficacy of drugs in relation to sex remains in need of intensive investigation.

### Limitations

Among the 27 drugs for which VB ratios were the only format of adverse event data, sex-biased PKs predicted sex-biased *VigiBase* ADR reporting ratios in 74% of instances (20 of 27 drugs were PK–VB ratio “concordant,” Table 2). In all but a few instances, *VigiBase* contained thousands of ADR reports, but as noted above, the number of men and women treated with each drug was not specified, nor are the links between the drug and specific ADRs known. It remains likely that some ADR sex differences in the *VigiBase* reflect unequal numbers of women and men treated with a given drug.

To definitively establish a cause effect relation between higher blood drug concentrations and ADRs requires concurrent measurement of PKs and ADRs in the same subjects. This has been accomplished infrequently (but see [56]).

Some have argued that what we ascribe to sex differences in the genesis of ADRs is causally related to differences in body weight. In reference to the sex difference in ADRs among patients taking zolpidem, Richardson et al. [57] stated “body weight, not sex, is the culprit.” They contend that body weight eliminates the statistical significance of sex as a variable in clearance of zolpidem, referencing a study by Greenblatt et al. [58]. However, the cited paper makes no such claim, instead stating that “AUC averaged 40% to 50% higher in females than in males receiving the same dose, and the sex effect was incompletely explained by body weight”. In a more recent review, Greenblatt et al. [59] note that women had on average 35% lower apparent clearance of zolpidem than men and that this difference was not explained by body weight. They conclude instead that the pharmacodynamic effects of zolpidem are greater in women due to a combination of higher plasma concentrations and greater intrinsic sensitivity [58]. The present analysis indicates that many sex differences in ADRs persist after corrections for body weight are made.

Lastly, while we appreciate the difficulties in conducting clinical research, the standards of evidence encountered in many reports cited herein were disappointing, especially so in the clinical trials submitted to, and approved by, the FDA: reports of no statistical differences between the sexes were routinely not supported by *F* statistics or *p* values, and were further complicated by small sample sizes and inflated type II error. Estimates of treatment effect size were also largely absent.

#### Recommendations

##### Recommendation #1

For all drugs currently in use, the FDA should post, on their website, PK data that were submitted as part of the drug approval process (i.e., NDAs) and associated peer reviewed publications. Generally, this information is unavailable for drugs approved prior to 2000. Once this information becomes accessible, scores if not hundreds of drugs with substantial PK sex differences will be revealed, which can facilitate corrective measures that will reduce female ADRs.

##### Recommendation #2

Credible evidence of sex differences in PKs should be made available in drug labels. In popular websites directed at the public either by drug companies, or by sites such as *WebMD*, the list of adverse effects should include known sex differences.

##### Recommendation #3

Almost all ADRs emerge from post-marketing reports, sometimes years after a drug appears on the market. The high correlation between elevated PKs in women and increased ADRs suggests that for drugs with higher female PKs, the initial dose should be lower for women than men and increased only if the lower dose fails to achieve the desired therapeutic effect. We anticipate this would eliminate or ameliorate many sex differences in adverse drug reactions. For orally administered drugs, pill splitting can begin to approximate a correction to the current practice where “one dose fits all.” Wherever feasible, drugs should be administered on a body weight adjusted basis (mg/kg) for men as well as women.

##### Recommendation #4

The FDA should require all empirical NDA data to be accompanied by documentation of statistical analyses which should meet requirements for publication in a peer-reviewed journal. Experiments reported in NDAs are less likely to be subjected to replication attempts by the scientific community, and therefore methodological or interpretive errors are less likely to be rapidly corrected by the iterative scientific process. Standards of evidence should be accordingly maintained high. At a minimum, this should include mandatory reporting of information on: a priori power analyses, measures of central tendency, variance, treatment of outlier data, statistical tests used to support statements regarding drug effects and lack thereof, specific *P* values, corrections deployed to control type I and type II error, and post hoc estimates of treatment effect magnitude. Such information is essential if clinicians are to develop informed decisions regarding sex differences in drug effects.

##### Recommendation #5

An appropriate understanding of the clinical relevance of sex-differences in drug treatment should be part of the board certification process for healthcare providers and reinforced in continuing medical education courses.

##### Recommendation #6

Establishing sex parity in the drug approval process should be explicitly identified as a long-term goal of the Department of Health and Human Services. The decades-long pattern of neglect of female animals in preclinical research and underrepresentation of women in clinical trials and research must be corrected, and the recent NIH oversight and vigilance must be maintained.

##### Recommendation #7

Pharmaceutical companies should pay more attention, beginning in the early drug development phases to sex-appropriate dosing [60]. Drug development commonly begins with pre-clinical modelling, in vitro experiments, and in vivo studies in mouse models. Recent analyses indicate that the overwhelming majority of basic research in mammalian physiology and pharmacology is based on work in male animals [6]. If, during the early stages of pre-clinical development, a compound is titrated or optimized specifically in male cells or in male mice, then any sex biases inherent in such models may be passed forward into later stages of drug development. The disproportionate expression of increased PKs and ADRs in women may be one result of this continued neglect.

## Perspectives and significance

The substantially elevated incidence of adverse drug reactions in women is undisputed, but the biological basis for this sex difference is poorly understood. The present study tested the hypothesis that sex differences in pharmacokinetics contributes to the female sex bias in adverse drug reactions. The analyses indicate that for many drugs administration of the same standard drug dose to women and men results in higher blood concentrations and/or longer elimination times in women and that these female-biased pharmacokinetics are a striking predictor of adverse drug reactions in women. Pharmaceutical pipelines that optimize drugs using males in vitro (male cells) and in vivo (male preclinical animal models) may contribute to the prevalence of these PK-ADR relations. Pervasive unintended and adverse drug responses may reflect routine overmedication of women for many drugs. Measures to ameliorate the sex difference in adverse drug reactions include reducing drug doses for women.

## Supplementary information

**Additional file 1:.** Supplementary Results.

## Data Availability

The datasets used and/or analyzed during the current study are available from either author and described in Additional file 1.
