# Supplementary material for: Sex differences in pharmacokinetics predict adverse drug reactions in women
Source: Biol Sex Differ. 2020 Jun 5;11:32. doi: 10.1186/s13293-020-00308-5 (PMC7275616; doi:10.1186/s13293-020-00308-5)
Supplement: Supplementary file 1 — Additional file 1:. Supplementary Results. [file 13293_2020_308_MOESM1_ESM.docx]

**Supplementary Results**

Sex-specific pharmacokinetics and adverse drug reactions

An abbreviated summary of the findings appears in Tables 1-3 of the main text. Pharmaceuticals are sorted by the WHO Anatomical Therapeutic Chemical (ATC) Classification System, which lists active ingredients of drugs according to the primary organ(s) or system(s) upon which they act, along with their therapeutic, pharmacological and chemical properties.

*ATC Classification A: Alimentary tract and metabolism*

Liraglutide. Women have 30% lower weight-adjusted clearance compared to men. Significantly more female than male subjects reported adverse events, most frequently headache, vomiting and nausea (1). No dose adjustments were suggested (NDA 022341).

Ranitidine. AUC and T_max_ were significantly lower for women than men (2). The incidence of duodenal damage in women at both four and eight weeks of treatment was significantly lower than that in men (3).

Rosiglitazone. A population PK analysis determined that net clearance in women is about 15% lower than for men (NDA 21071; Avandia). From a meta-analysis of 10 randomized controlled trials and two large observational studies, Loke et al. (4) concluded that rosiglitazone and pioglitazone were associated with a significantly increased risk of fractures; in five randomized controlled trials this risk was greater among women but not among men.

Ondansetron. Women cleared ondansetron more slowly than men, producing higher absolute bioavailability (5). The AUC was >2 fold higher in women than men (354 vs. 154 ng·hr/ml), and the C_max_ was 83 ng/ml for women vs. 51 ng/ml for men. Although the FDA-approved labeling accurately identified this sex discrepancy in plasma concentrations and clearance, no sex-aware dosing adjustment is recommended (Zofran, FDA product labeling; (6)). The *VigiBase* ADR ratio was 2.14, based on 16266 reports.

*ATC Classification B: Blood and blood forming organs*

Heparin. After a standard heparin bolus and infusion, women had significantly higher values than men (0.56 vs. 0.35 units/ml; (7). Sex differences were reported in 273 out of 621 adverse events associated with heparin. Heparin-induced blood and lymphatic system disorders were greater for female than male patients. Drug labels indicate that a high risk of bleeding after heparin treatment is increased in women older than 60 years. After matching for age, body mass index, and type of antithrombotic therapy (e.g., heparin), bleeding risk after percutaneous coronary intervention remained significantly higher in women than men. Bleeding was associated with increased risk of 1-year mortality (8). Gutie´rrez-Chico et al. (9) noted a higher incidence of bleeding complications from antithrombotic drug treatment in women that might be related to frequent over-dosage.

Aspirin. Aspirin is frequently utilized in treating cardiovascular disease. Oral bioavailability of aspirin was higher in females than males, with significantly greater plasma concentrations in women (10–13). The AUC and elimination *t*_1/2_ of aspirin were significantly greater, and aspirin plasma hydrolysis rate significantly lower in women. Aspirin clearance was 61% higher in men than in women (14).

Aspirin is much more effective in preventing myocardial infarction in men than women (42% vs. 19%; (15–17)). The use of aspirin is associated with a higher reduction of risk for ischemic stroke in women (17). Aspirin administered to men produced a nonsignificant increase in stroke. In another report, aspirin administered to women did not significantly reduce the risk of major cardiovascular events, but significantly decreased risk of stroke (18). Fibrinogen concentration in aspirin-treated women is significantly higher than in men and may contribute to higher platelet aggregation (19). Women have lower mean percent inhibition of platelet aggregation and greater prevalence of aspirin resistance than men (20). Inhibition of platelet aggregation may be insufficient in women; female patients might benefit from higher maintenance doses (21). Young women exhibit greater post-treatment reactivity while on aspirin, which may contribute to the excess risk observed in this population (22). An increased risk of non-fatal/fatal cardiovascular disease associated with aspirin treatment in patients with Type 2 diabetes was present in women but not in men (23). Analyses of sex differences in bleeding from antithrombotic treatment with aspirin yielded conflicting results. No significant difference in bleeding risk between women and men on aspirin was found in one study, a higher bleeding risk was seen in men in another study and in a meta-analysis no sex difference in bleeding risk was found (24).

Warfarin. Sex was a significant independent contributor to warfarin clearance with women having 22% lower S-warfarin clearance than men (25) as also reported by Takahashi et al. (26). Studies cited by Whitley et al. (27) consistently report lower warfarin doses are required for women compared with men, e.g., median warfarin daily dose requirements varied significantly with sex and were 2.9 mg for women and 3.7 mg for men (28). Garcia et al. (29) note that women required 4.5 mg less warfarin per week; the recommended initiation dose of 5 mg/day will be excessive for 82% of women and 65% of men. Major bleeding complications occur more often in women than men treated with warfarin and thrombolytics for venous thromboembolism (30). Women on warfarin were 3.35 times more likely than men to experience a major bleed (31).

Clopidogrel. The C_max_ was 1.7 fold higher and the AUC 1.88 times higher in female than male patients (22). A low dose of this blood thinner increased fracture risk in women but fracture incidence for men was only increased at higher doses (32). Clopidogrel is associated with higher incidence of gastrointestinal symptoms in women with irritable bowel disease symptoms (33) with a higher prevalence of bleeding reports in women (24). Young women have greater baseline clotting tendency, reduced response to clopidogrel, and greater post-treatment reactivity which may contribute to the excess risk observed in young women (22).

Dabigatran. The AUC is 20-30% higher in elderly women than elderly men (34). Dabigatran was similarly effective for stroke prevention in both sexes, but it decreased the risk of major bleeding in men but not in women (35) and a stratified analysis suggests increasing odds of dabigatran-induced adverse bleeding as age increases among women (36).

Icatibant. Younger women exhibited lower clearance rates with 2.3-fold increases in both C_max_ and AUC compared to men (NDA 022150). In elderly females, C_max_ was similarly increased 2.3-fold compared to age-matched males, and AUC was 1.8-fold higher. Nevertheless, no dosage reductions are recommended for women on the drug label, justified based on post hoc PK analyses from two additional trials and a population PK analysis of all available data. The ADR ratio was 3.18, based on 1765 reports.

*ATC Classification C: Cardiovascular system*

Over the past 4 decades the incidence of cardiovascular diseases has been reported to progressively decline in men, but it has continued nearly unchanged in women (37). HMG-CoA reductase inhibitors (statins) are a frontline treatment protocol for lipid management; statins interfere with the action of HMG-CoA reductase, a key enzyme in the synthesis of cholesterol. The benefit of statins in women for secondary prevention has been demonstrated but, their role in primary prevention of cardiovascular disease remains controversial (38). Clinical trials on statins have been conducted almost entirely on men (39,40). Because statins have not been adequately tested in women, treatment decisions such as drug selection and dosing regimens for women are potentially dangerous (41). In addition, women are more prone than men to develop *torsades de pointes* (TdP), a life-threatening cardiac rhythm disturbance during administration of multiple cardiovascular drugs that prolong cardiac repolarization (42).

Torasemide. Both absolute and body weight-adjusted AUC and C_max_ values were 30% to 40% higher in women than men, *t*_1/2_ was significantly longer and oral clearance was slower in women (43). These sex differences are accompanied by a higher rate of major ADRs in women; a pharmacovigilance project indicated that 66% of hospitalizations due to torasemide ADRs occurred in women, a number that was not accounted for by sex‐specific differences in drug prescription rates (43).

Pravastatin. Among patients with a specific hepatic transporter molecule genotype (SLCO1B1 c.521TT genotype) women administered a single dose of pravastatin had a 147% greater peak concentration in plasma over the next 12 h, and a 142% greater AUC than men (44). The incidence of coronary heart disease was more pronounced in older women than in older men (45).

Amlodipine. In middle-aged and older patients, clearance was ~30% faster in women than men (46). Adverse reactions are substantially more common in women for edema, flushing and palpitations (Norvasc; product label).

Digoxin. Clearance is 12-14% lower in women than men (47). The original digoxin trial suggested increased mortality among women. Death from any cause was 5.8% higher in women (48) perhaps reflecting higher drug concentrations (49). Digoxin therapy is associated with an increased risk of death from any cause among women, but not men (50). It has been suggested that patient sex should be considered when prescribing this drug (51,52).

Verapamil. Clearance of oral verapamil was accelerated in women (53); the *t*_1/2_ of verapamil and mean residence time were significantly shorter in women than men (54). After administration of sustained-release verapamil, however, oral clearance was lower in women than men, 43 vs. 75 ml/min/kg, with a similar sex difference after regular-release verapamil. This sex difference was not evident when verapamil was administered intravenously, suggesting that intestinal processes likely influence sex-specific differences in drug clearance (55). Constipation, edema, fatigue and headache occur more frequently in women than men (56) treated with controlled-onset, extended-release verapamil.

Aliskiren. The AUC was 22% lower and C_max_ was 24% lower in men than women, but sex differences were abolished when corrected for body weight (57). In women increases in diarrhea rates were evident starting at a dose of 150 mg daily, comparable to those seen at 300 mg for men.

Losartan. C_max_ was 100% higher and AUC was 250% higher in women than men. Despite these differences no dose adjustment was recommended for women ((58), citing drug prescribing information from the *Physicians Desk Reference* and Novartis). Os et al. (59) reported that women had more adverse events but fewer serious drug-related adverse events than men, but none of these differences was statistically significant. Men on losartan were more likely to be hospitalized for angina and tended to have overall higher mortality (3.6% vs. 1.8%; (59)).

Propranolol. Oral clearance was 63% higher in men than women (60) and AUC and C_max_ in women were 74% and 99% higher, respectively, than in men (61). The incidence of dizziness, muscle pain, headaches and dry mouth was substantial higher in women than men (62).

Dofetilide. Women’s systemic exposure is 14–22% higher after correcting for body weight and creatinine clearance (FDA label; Roukoz and Saliba, 2007). The risk of TdP in women was approximately three-times that in men and directly related to plasma concentration and dose (63). Modification of doses based on creatinine clearance and baseline QT_c_ measurements can accommodate sex-specific dosing of dofetilide.

Pitavastatin. Among healthy subjects C_max_ and AUC were 60 and 54% higher, respectively, in women than men without affecting efficacy or safety in clinical studies (NDA 22363). The ADR ratio was 1.12, based on 5003 reports.

Labetalol. For women receiving similar average dosages and having similar body weights, dose-corrected concentrations were 80% higher in women, and C_max_ was more than 2-fold higher in women, but the drug effect was similar in both sexes, as the protocol dictated dosage titration to a specific antihypertensive effect. Women required nearly 2-fold higher drug concentrations to achieve a similar antihypertensive effect (64). ADR ratio of 1.5, based on 4210 reports.

Nifedipine. Clearance was significantly slower in men than women 9.3 ± 0.6 versus 12.1 ± 1.5 ml/min/kg (65). The ADR ratio was 1.29 based on 36924 reports.

Metoprolol. Women had significantly higher C_max_ and AUC values than men after oral treatment with metoprolol tartrate; women also exhibited a greater reduction in exercise heart rate and systolic blood pressure (66). Sharma et al. (67) observed similar sex differences when metoprolol was administered in the presence of a steady state of diphenhydramine, which increased the metoprolol AUC by 84% in women and only 45% in extensive metabolizer men. Sex differences in PKs persisted after correction for differences in body weight. Ueno and Sharma (68) suggested that women may require lower-than-standard doses to avoid adverse effects, whereas men may require larger doses to obtain therapeutic benefit. On the other hand, Cocco and Chu (2006) reported that despite presumed greater plasma concentration of metoprolol in women, there was a significant difference in anti-ischemic effect in favor of men. ADR ratio of 1.26, based on 39751 reports.

*ATC Classification G: Genito-urinary system and sex hormones*

Mirabegron. The C_max_ and AUC for treatment of overactive bladder were approximately 40-50% higher in females than males; systemic exposure was 20-30% higher in women after correction for body weight differences. Mixed results were reported in studies that examined the effect of sex on discontinuation of treatment. In Canada and the Czech Republic mirabegron persistence rates were higher in men than women (Table 1; (69,70)) whereas the reverse was found in an UK-based studies (71) and no sex difference in a Japanese study (72). There are no label indications of this difference and no recommendations that dose adjustments are necessary based on sex (NDA 202611).

Darifenacin. Darifenacin hydrobromide clearance was 31% lower in women than men (73); C_max_ and AUC at steady state were approximately 57-79% and 61-73% higher in females than males, respectively; women reported more adverse effects than men (74) but no dose adjustment was recommended based on sex (NDA 021513).

Trospium. Trospium chloride dosed to elderly men and woman generated AUC and C_max_ values 26% and 68% higher, respectively, in women and *t*_1/2_ was prolonged by approximately 4 h in women (cited by (75)). Women were twice as likely as men to develop cognitive impairments (76) but sample sizes were small.

*ATC Classification H: Systemic hormonal preparations*

Prednisone. Prednisone is converted to prednisolone by the liver before it becomes active. Body weight–normalized free prednisolone oral clearance was 22% higher in white men than in white women, and 40% higher in black men than black women (77). In stable renal transplant recipients, median dose- normalized total prednisolone AUC was significantly higher in women than men (78). Prednisone ADRs were significantly more common in women who also reported significantly more intolerable ADRs (depression, fatigue, hair loss, mood swings, moon face, sleeplessness, stomach complaint, and weight gain) and were less willing to accept a dose increase compared to men (79).

Methylprednisolone. Women in the luteal phase of the menstrual cycle cleared methylprednisolone significantly faster and had a shorter elimination half-life than men when values were normalized to ideal body weight (IBW) and the AUC was significantly lower in women; the concentration of methylprednisolone sufficient to suppress endogenous cortisol was >10-fold lower in women (0.11 vs. 1.69 ng/ml; (80); the authors suggested that men and women should receive the same methylprednisolone dose normalized to IBW because even though women eliminate the drug faster, they also have a greater response to a lower concentration of the drug. The VigiBase ADR sex ratio was 1.39, based on 35227 reports.

*ATC Classification J: Antiinfectives*

Levofloxacin. Women had a 24% greater exposure after receiving a 500 mg i.v. dose with a significantly smaller steady-state V_d_ and a slower clearance time than men (81). Fixed i.v. doses produced greater drug exposure in women and an increased risk of fluoroquinolone toxicity. Dosage adjustments based on sex should be considered on an individual basis (81).

Erythromycin. Women cleared erythromycin significantly more rapidly from the serum than did men (82). Significantly more women than men develop TdP arrhythmias after treatment with erythromycin (83).

Voriconazole. The NDA indicated that the C_max_ and AUC for young women were 83% and 113% higher, respectively, than in young men; these sex differences were no longer present in subjects >65 years of age. In the same document, oral dosing generated AUC values 45% higher in women than men, with comparable C_max_. Steady state trough concentrations (C_min_) were 100% and 91% higher in women treated with tablet and oral suspension, respectively. Nevertheless, no indications were included on the approved drug label to suggest that dosage adjustments based on sex should be considered (NDA 021266). Females on voriconazole exhibited a greater increase in cardiac QTc symptoms than males.

*ATC Classification L: Antineoplastics and immunomodulating agents*

Cyclosporine. Multiple studies document that women clear cyclosporine 30–88% faster than men (reviewed in (52)). In psoriasis patients, the incidence of adverse events was 37% higher in fertile women and 18% higher in postmenopausal women than in age-matched men, but differences were not statistically significant (84).

Fluorouracil. Men had a 26% higher elimination of 5-fluorouracil and women had a significantly higher AUC than men (85). Women experience significantly greater and more frequent and more extensive toxicity (stomatitis, leukopenia, alopecia, diarrhea) with fluorouracil-based chemotherapy (86,87)**.** Women also experienced more frequent moderate to severe mucositis than men (88).

Paclitaxel. Elimination was 20% higher in men than women with solid tumours (89). In women treated with paclitaxel-eluting stents myocardial infarction was slightly worse as compared with their male counterparts (90). In expanded-use patients, women had signiﬁcantly higher death rates and total lesion revascularization (91).

Capecitabine. The AUC of the capecitabine metabolite FBAL was 37% greater in women (92). The AUC of capecitabine was 87% greater in females than males. Women had a significantly higher dose-limiting toxicity incidence than men (68 vs. 52%; (93)).

Infliximab and adalimumab. Clearance of infliximab was about 33% lower in women than men (94). The mean clearance across all doses of adalimumab was significantly lower in women than men. The 30% difference appeared to be due mainly to 23% differences in body weight (95). During treatment with anti-TNF antibodies women were more at risk for allergic reactions and subsequent discontinuation of the treatment than men (96); specifically, the most frequent adverse reactions to infliximab and adalimumab were allergic responses (15% of all infliximab users and 7% of all patients treated with adalimumab) and for both agents a significantly higher rate of allergic reactions occurred in women (96).

*ATC Classification M: Musculo-skeletal system*

Ibuprofen. Sex differences were observed in weight-adjusted pharmacokinetic parameters of both *R*- and *S-*ibuprofen. Women had a significantly lower *R*-ibuprofen C_max_ and *t*_1/2_ and *S*-ibuprofen AUC and C_max_, and a higher *S*-ibuprofen clearance rate than men. These differences may be related to CYP2C8 or CYP2C9 genotypes; a reanalysis of the association between ibuprofen pharmacokinetics and sex, restricted to wild-type subjects, found significantly lower *R*-ibuprofen C_max_ and *t*_1/2_, and *S*-ibuprofen C_max_ and AUC in women and a higher ibuprofen clearance in women. A robust sex-specific difference in increased drug elimination times, may decrease drug efficacy and necessitate an increase in drug doses for women, which may be accompanied by increases in ADRs. The phase of the menstrual cycle did not affect either *S*-ibuprofen or *R*-ibuprofen pharmacokinetics but women treated with oral contraceptives had lower AUC and higher clearance than women not contracepted (97). Conflicting results reporting no ibuprofen differences in pharmacokinetics were based on sample sizes of 8 per group (98).

An ibuprofen dose of 800 mg was effective in young men but did not produce analgesia in young women in an experimental pain test (99,100). This may reflect lower *R*-ibuprofen *t*_1/2_ and *S*-ibuprofen AUC and C_max_ in women than men and suggests that women may require higher ibuprofen doses to achieve comparable analgesia. ADR of 1.46 in 130176 reports.

*ATC Classification N1 and N2: Nervous system – anesthetics and analgesics*

Women emerge faster from general anesthesia than men, but their recovery trajectory is significantly poorer, in terms of higher pain scores and need for treatment of nausea and vomiting (101). Women had higher bispectral index values (BIS; an indicator of depth of anesthesia) than men, despite being administered similar drug amounts and having a faster time to eye opening. Women were less sensitive to the hypnotic effects of multiple anesthetics (101).

A review of several studies of patient-controlled analgesia (PCA) with different opioids revealed significantly greater analgesia in women than men. Indeed, the longer the duration of PCA, the greater the sex difference (102). The lower opioid consumption among women reported in PCA studies after surgery may partially reflect the greater frequency of side effects among women (see below; (103)). Elderly women had higher incidences of nausea, vomiting, vertigo, inappropriate sensory and motor blockages, as well as experiencing more pain in bed rest and daily activities during PCA. Elderly men had more pain experience while coughing but had higher satisfactions to PCA (104).

Morphine. Peak plasma concentrations were 25% higher in women than men, but this difference was eliminated when data were corrected for body weight (105). In terms of the intended drug response to morphine and other opiates, repeatable sex differences exist in opiate-induced analgesia. Sarton et al. (106) documented a slower speed of onset and offset in morphine-induced analgesia, but a greater potency in women. Similar results were obtained for pain threshold and pain tolerance. Women experienced more severe postoperative pain and required a greater dose (+11%) of morphine than men in the immediate postoperative period. This sex-related difference was not present in elderly patients (107).

Sex differences in ADRs to opiates are widespread. Respiratory depression is a greater risk in females: a bolus of 100 µg/kg morphine, followed by a continuous infusion of 30 *µg*/kg/h, reduced the slope of the ventilatory response to CO_2_ in women but not in men. Morphine decreased hypoxic sensitivity in women but not in men. Neither inclusion of body surface area, nor correction for body fat by using it as a covariate eliminated these sex differences in morphine-induced respiratory effects (108).

•

Morphine attenuated cardiovascular reactivity in an ischemic pain task in men but not in women; women were also significantly more likely to experience emesis and nausea after taking morphine (109), and more likely to manifest respiratory depression after morphine treatment (108). Following a dose of 0.08 mg/kg morphine (i.e., corrected for body weight) women reported greater frequency of negative side effects than men (110). Women report feeling more ‘spaced out’, ‘heavy’ and ‘sluggish’ than men after comparable morphine treatments (111). The incidence of respiratory depression (52% vs 32%) and postoperative nausea and vomiting (43% vs 4%) were higher in girls than boys with perioperative morphine doses ≥0.3 mg/kg (112).

Oxycodone and related opiates. Plasma OxyNorm (oxycodone) concentrations are 25% higher in women than men, even when adjusted for body weight, (OxyNorm product information). The product insert states that “…prolonged use of opioids may result in impairment of reproductive function, including fertility and sexual dysfunction in both sexes, and irregular menses in women,” (p. 4), yet incongruously concludes that “…no significant male/female differences [were] detected for efficacy or adverse events in clinical trials.” (p. 6) Women were more sensitive to the positive subjective effects of oxycodone than men (113). Women also experienced significantly more oxymorphone-related nausea (24% vs. 12%) and pruritus (9% vs. 5%) than men, and in oxycodone-experienced patients, successful titration for chronic low back pain was achieved less frequently by women compared with men (35% vs. 56%; (114)). Functional impairment, psychiatric severity, and higher likelihood of using opioids to cope with negative affect and pain were all significantly greater in women than men for oxycodone and hydrocodone (115).

Buprenorphine. Women have signiﬁcantly higher AUC (58.4 vs. 41.9 ng·h/ml) and C_max_ (6.97 vs. 5.15) values (116). Women had a signiﬁcantly greater likelihood of reporting sleep disturbances than men (117).

Tramadol. A single oral dose induced a 12% higher peak concentration and a 35% higher AUC in women than men (product information for Tramal). The risk of treatment cessation in response to Tramal, due to emergent adverse reactions was 2.2 times higher in women than men (118).

Zolmitriptan. After oral dosing for the treatment of acute migraine, C_max_ and AUC were ~2 fold higher in women than men (119,120), which may put women at increased risk for toxicity when administered the same maintenance dose as men (121). More women than men reported symptoms of pressure and tightness usually of the face and neck (119).

Ketamine. Sigtermans et al. (122) reported a 20% greater elimination clearance of S(+)-ketamine and S(+)-norketamine in women than men, resulting in higher drug plasma concentrations in men. They did not detect any sex differences in several pain tests, but men showed a greater performance decrement after ketamine administration compared to women on the Hopkins Verbal Learning Task and a greater subjective sense of memory impairment than women (123).

Lignocaine (lidocaine). The percentage of lignocaine unbound in the plasma of women taking oral contraceptives was significantly greater by 17% than in men (124). In one clinical report, 64% of adverse reaction reports were from women, for a female: male ratio of 1.78, but it is unclear whether or not this ratio merely reflects the frequency of lignocaine use in men and women (125). ADR reports in *VigiBase* were similarly sex biased towards women (1.64 ADR ratio, based on 21583 reports).

Propofol. After a single bolus, women had significantly higher values of weight-corrected AUCs of propofol glucuronide (1.25-fold), 4-hydroxypropofol-1-glucuronide (2.1-fold) and 4-hydroxypropofol-4-glucuronide (1.7- fold; (126,127)). However, women also emerge from propofol anesthesia significantly faster than men, with plasma concentrations of the drug declining significantly more rapidly in women (128). Women require more propofol than men to maintain the equivalent BIS index level under general anesthesia. Women treated with an augmented sedative cocktail of propofol / remifentanil / dexmedetomidine consumed comparable amounts of morphine compared to men, but when drug ingestion was normalized for body weight, they consumed much more morphine than men (129). ADRs were more frequently reported for women than for men (1.45 ADR ratio, based on 20903 reports).

Butorphanol. The AUC and *C*_max_ values for single intranasal administration were significantly greater by 23–29% in women versus men for three doses of the drug (130). Nalbuphine and butorphanol significantly prolonged the duration of analgesia in women compared with men (131). ADR ratio of 3.23, based on 5475 reports.

Paracetamol (acetaminophen). Miners et al. (14; see also (132)), reported that clearance was 22% lower in women than men but Court et al. (133) found that total or metabolite clearance values were not different between sexes; however, significantly shorter plasma half-life values were observed for acetaminophen, acetaminophen glucuronide, and acetaminophen sulfate in women versus men. 71% of patients admitted to hospitals for acetaminophen overdose were women (134,135). ADR ratio of 1.68, based on 125775 reports.

*ATC Classification N3 and N4: Nervous system – antiepileptics and anti-Parkinson’s*

Carbamazepine(s). Absolute clearance was higher and *t*_1/2_ shorter in women than men (136). The potential toxic dose of oxcarbazepine was substantially lower for women than men (1200 vs. 1800 mg) as also was true for carbamazepine (800 mg for women vs. 1200 mg for men; (137)). Thus, women may benefit from lower doses given their tendency to reach toxic concentrations at lower doses (137) although the opposite recommendation has been made; women may require an increase in milligram per kilogram dosing if plasma levels do not correspond to the prescribed dose (138). Carbamazepine and oxcarbazepine are associated with cognitive impairment which is greater in women (139). High and low density lipid differences were significantly more pronounced in women than men treated with carbamazepine (140).

Gabapentin. C_max_ was significantly different between sexes: ~25% higher for women than men (141). In data combined across two phase III and one phase IV studies of gastroretentive gabapentin (G-GR), the most common adverse events were dizziness, somnolescence and nausea. The probability to report any ADR was 0.6 for females and 0.4 for males, and only female sex was a significant predictor of ADR reporting: correcting for other covariates, women were 1.9 times more likely to report ADRs at any time after treatment (142).

Perampanel. Healthy women had 32% higher AUC than men; oral clearance was 17% lower in women than men. Vazquez et al. (143) suggested that lower clearance and corresponding increase in plasma concentrations in women may account for the slightly greater seizure reduction seen in women. Common adverse events such as dizziness and headache occurred more frequently in women than men. ADRs were the main reason for discontinuation of treatment, with a higher percentage for women (10.9%) than men (6.8%). No dosing reductions were recommended for women in labels.

Pramipexole. For this dopamine receptor agonist administered for the treatment of Parkinson's disease, the steady-state C_max_ and AUC following a single dose were 28-32% and 35-43% greater in women than men, respectively (144). No sex difference with respect to Parkinson’s treatment, but nausea and fatigue were more frequently reported by female than male restless leg syndrome patients (NDA 020667).

Ezogabine (retigabine). AUC values were 20% higher in young women than young men and 30% higher in elderly women than elderly men. The C_max_ values were approximately 50% higher and approximately 100% higher in elderly and young women than men, respectively. There was no sex difference in weight-normalized clearance (145); dosage adjustments on the basis of sex are not considered necessary as ezogabine is titrated based on individual patient efficacy and tolerability ((146) citing data from drug manufacturer Glaxo). ADR was 0.90, based on 582 reports.

*ATC Classification N5: Nervous system – Psycholeptics*

In general, patients on long-term antipsychotic therapy, with doses adjusted according to therapeutic efficiency, exhibited hyperprolactinemia that was more frequent and occurred at a lower daily dose in women, despite men being administered, on average, a two-fold higher daily dose than women (147).

Marazziti et al. (2013) noted that because blood volume is less in women and lipid mass is greater than in men; the V_d_ of lipophilic drugs, such as antipsychotics, is greater in women than in men, which may prolong the half-life of antipsychotics in women. This observation is congruent with the findings summarized below.

Clozapine. Clozapine is in widespread global use and on the WHO list of essential medicines. Norclozapine is its major active metabolite. Clozapine and norclozapine concentrations in the circulation after comparable dosing regimens were 35% and 36% higher, respectively, in women, after controlling for other variables (148–150). Fabrazzo et al. (1996) reported that clozapine plasma concentrations are twice as high in women than men during early phases of treatment (4 - 6 weeks), but not after 24 weeks. The dose-adjusted concentrations in women were significantly (28%) higher than in men. Metabolic and gastrointestinal ADRs are common in patients taking clozapine, and sex biases in ADRs are symptom-specific. Metabolic syndrome was reported to be more prevalent in men on clozapine (151), although a report with a larger sample size indicated a female bias in clozapine-associated metabolic syndrome (152). In a meta-analysis focused on case reports, West et al. (153) concluded that clozapine-induced gastric hypomobility was more common in men; however, other reports indicated that women taking clozapine were more likely to develop ileus (154), and another report indicated that sex did not impact colonic transit time in patients on clozapine (Every-Palmer et al., 2016). In a broader meta-analysis, Alberich et al. (2019) identified elevated blood glucose, laxative use, obesity, increases in body mass, type II diabetes, neutropenia and leukopenia as ADRs that were increased in women, and hypertension, increases in BMI, homocysteine, increased basal metabolic rate, increased plasma lipids, QTc, blood dyscrasias, myocarditis and cardiomyopathy as male-biased ADRs. Perhaps the most consistently-reported ADR on clozapine is weight gain. (155) identified female sex as a risk factor for significant weight gain among 110 schizophrenics on clozapine, data which confirmed a previous report indicating greater weight gain in women on clozapine (156).

Olanzapine. In a psychotic inpatient population on a stable olanzapine dose, adjusted mean plasma concentrations were 18.5 ng/ml for men and 31.7 ng/ml for women. Weight-corrected concentration/dose ratios for women were 33.5% higher than those shown by men (157); clearance from the blood is ~30% reduced in women than men (NDA 20592). However, sex did not significantly affect plasma olanzapine concentrations in an adolescent study population (158). Female sex was significantly associated with probability of undesired or severe weight gain (159,160). Olanzapine labeling recommends lower doses in patients in whom higher exposures are anticipated (161). A pharmacometric analysis suggests that for olanzapine to achieve 70% dopamine (DA) receptor occupancy, men require ~20 mg/day and women require only half that amount, which suggests clinicians should consider this difference in selecting starting doses (162).

Risperidone. Dose-adjusted plasma concentrations were 18.7% higher in women (158,163). Another study indicated 30% higher serum concentrations in women compared to men (164,165). Hyperprolactinemia, a neuroendocrine side effect of tuberoinfundibular DA inhibition, was significantly greater in women than men treated with risperidone (127 vs. 54 ng/ml; (166,167)). Hyperprolactinemia is a well-established risk factor in the development of mammary carcinomas (168). Women treated with risperidone also exhibited neurological effects more frequently than men (169); sex differences in risperidone-associated ADRs also included headache and hypotension, which were significantly more frequent in women than men (31% vs. 11%, and 17% vs. 0%, respectively (170)).

Aripiprazol. C_max_ and AUC are 30-40 % higher in women than men and the apparent oral clearance is lower in women (Figure 23 in NDA 021436). Aripiprazol treatment is accompanied by sex differences in blood pressure, heart rate and an elongated corrected QT (QT_c_) interval; women and patients with higher AUC and C_max_ values were also more prone to gastrointestinal ADRs (171). Nevertheless, no dosage adjustment is recommended based on sex (NDA 021436). Indeed, the NDA repeats the clause “Examination of population subsets (age, race, and gender) did not reveal any differential responsiveness on the basis of these subgroupings.”, and variants thereof, in multiple instances, without indicating the statistical basis of these conclusions (significance level for rejection, statistical trends, sample sizes, power, etc.).

Diazepam. Data indicating robust sex differences in circulating diazepam were available in the 1970’s: the mean percent of unbound pharmacologically-active diazepam was higher in women than men (10.8% vs. 5.8%, (172)). Sample sizes were small and this nearly two-fold difference only approached statistical significance. In contrast, in another report clearance of total (bound + free) diazepam was substantially higher in older women than men (0.48 vs. 0.24 ml/min/kg; (173)). MacLeod et al. (174), again with small sample sizes, noted that the *t*_1/2_ of diazepam was significantly greater in women than men (46.2 h vs. 32.0 h). Moreover, female sex hormones markedly affect the clearance: women using contraceptives exhibited significantly greater percent of diazepam free in plasma as compared to contraceptive-free women, both of which were greater than male percentages (174). These data are consistent with ADR observations that indicate that women are more impaired than men in psychomotor skills after diazepam administration (175).

Zolpidem. Zolpidem was FDA-approved in 1992 to treat insomnia (NDA 19908). The sole pre-approval assessment of the effect of sex on zolpidem PKs enrolled 19 women and 49 men; both the C_max_ and AUC were ~45% higher in women. Peak concentrations were also reached 1 hour sooner in women. The lack of specific details of study design did not convince the FDA to recommend dose reductions for women. This was a missed opportunity, which would be repeated many times over the next quarter century. Eight hours after a 10 mg dose of zolpidem, 15% of women and 3% of men had blood zolpidem concentrations of 50 ng/ml or greater (176). Fast forward to 2011, when more rigorous pre-approval testing of a newer form of zolpidem tartrate (Intermezzo) was FDA-approved (NDA 022328) after enrolling 259 women and 118 men. Women cleared zolpidem tartrate after sublingual administration of a 3.5 mg dose at a significantly lower rate than men (2.7 ml/min/kg vs. 4.0 ml/min/kg); C_max_ and AUC parameters again were ~45% higher in female subjects (e.g., (177)), replicating the zolpidem NDA assessment 21 years prior. As recently as 2007, labeling information recommended a dose of 10 mg zolpidem immediately before bedtime for both women and men. By 2013, prompted by post-marketing reports of cognitive deficits, the label advised an initial dose of 5 mg for women. Such reports included data indicating that women were more seriously compromised than men in driving performance on the morning after middle-of-the-night administration of 10 mg zolpidem (178). In a study that did not test for sex differences Yang et al. (179) reported that use of zolpidem on the previous day might be associated with an increased risk of motor vehicle accidents. By 2013 the label information for extended release zolpidem tartrate (NDA 021774) finally advised using the lowest effective dose for the patient, based on findings that women clear zolpidem tartrate from the body at a lower rate than men, with C_max_ and AUC approximately 50% and 75% higher at the same dose in women, 6 to 12 hours after dosing (i.e., on the following day). The recommended initial dose of Ambien CR for adult women was 5 mg, and for men 5 or 10 mg. This change in labeling precipitated an increase in the percentage of patients prescribed low-dose zolpidem that was only significant for young women (180). It also did not escape pushback from scientists and the pharmaceutical industry: Greenblatt et al. (2019) in a review and reanalysis of earlier studies, noted that none of the epidemiological studies demonstrating driving deficits after zolpidem treatment considered the issue of timing of treatment dosage relative to the timing of driving tests and concluded that there is no established relationship between zolpidem concentration and driving impairment. They recommended that the FDA revise the mandated dose recommendations for zolpidem to become equivalent to doses in men. They also suggest that dose reductions in women may negatively impact treatment of insomnia, echoing the drug sponsor’s concerns after the label change was proposed by the FDA. These are valid considerations; earlier data, however, not associated with driving tests, provide circumstantial evidence that zolpidem concentrations would be substantially increased at times corresponding to when driving could reasonably be assumed to be occurring: i.e., 12 hours after dosing, zolpidem concentrations were 2- to 3 fold higher in adult female compared to adult male subjects (NDA 021774). And 8.5 hours after a dosing of 10 mg Ambien, circulating concentrations averaged 38 ng/ml in women and 17 ng/ml in men; 5 of 16 women, but only 1 of 17 men generated blood levels ≥50 ng/ml (NDA 021774). Greenblatt et al. (2019) reanalyzing data from a study that did not evaluate sex differences (181) reported that 8-hour post-dosage plasma zolpidem concentrations were higher in women than in men for both the 10 mg immediate release and 12.5 mg modified release dose trials and that self-rated and/or observer-rated sedation was significantly greater in women than men for both treatments.

Clobazam. Distribution was larger in women than men of all ages. In young subjects, elimination *t*_1/2_ was longer in women than in men (182). ADR ratio of 1.1 based on 6372 reports.

Eszopiclone. Under steady-state conditions, women had a 25% higher AUC for eszopiclone (NDA 21476). Dysgeusia was more intense and longer lasting in women than in men, and positively correlated with drug plasma and saliva concentrations (183).

Sertindole. Multiple oral dosing yielded C_max_ values 20% higher in young and 31% higher in elderly women compared to men of similar age. The mean AUC values within a single dosing interval were 29% higher in women than men and persisted after correction for body weight; this sex difference may reflect an age disparity as women in the study were substantially older than men (184). ADR ratio of 1.06, based on 611 reports.

Thiothixene (tiotixene). Orally administered thiothixene had higher clearance in men than women (48 vs 22 L/min; (185)). The ADR ratio was 1.35, based on 949 reports.

Oxazepam. Clearance of oxazepam was significantly slower in women than men (9.7 h vs. 7.8 h; (186)). The ADR ratio was 1.31, based on 6573 reports.

Temazepam. The elimination *t*_1/2_ was consistently longer in women than men (16.8 vs. 12.3 h); clearance of unbound temazepam was higher in men than women (187). The ADR ratio was 1.34 based on 4508 reports.

Zopiclone. The C_max_ was 14% to 24% higher, and the AUC was 16% to 20% higher in women than men (188). Zopiclone impaired driving equally in men and women in the morning, until 11 hours after bedtime ingestion (189); however, these behavioral conclusions were based on small sample sizes (13 men, 12 women) and were performed in patients with no history of insomnia. The ADR ratio was 1.55, based on 9632 reports in the *VigiBase*.

*ATC Classification N6: Nervous system – Psychoanaleptics*

Antidepressants are disproportionately prescribed to women. During 2011–2014, 12.7% of persons in the U.S. aged 12 and older reported taking antidepressants in the previous month; the overall ratio of women to men on antidepressants is at least 2:1 among Caucasians and Hispanics; this ratio increases to ~2.5:1 among non-Hispanic Asian Americans and non-Hispanic African Americans (190).

Imipramine. A meta-analysis of 12 studies found that women had significantly higher dose-adjusted plasma concentrations than men, although in three of the studies the adjustment of dose to body mass eliminated the effect of sex (191). Men exhibited better responses to imipramine than pre-menopausal women (192). Depressed women responded significantly more slowly than men to imipramine (193). Baca et al. (194) reported that women treated with imipramine experienced significantly more ADRs including dry mouth, constipation, sweating and tremor. The proportion of patients who dropped out of imipramine drug trials due to ADRs was 27.8% for women and 11.5% for men (194).

Nortriptyline. Female patients do not clear nortriptyline as fast as male patients do; plasma concentrations were significantly greater in female than male patients (3.8 ± 1.1, versus 2.1 ± 0.5); this difference manifested despite a combined total study sample size of 21 patients (195). In a clinical trial, a significant increase in ADRs, self-rated dry mouth, was present during all 6 weeks of nortriptyline treatment in women, whereas in men this symptom was only manifest on two of the study weeks (196).

Fluoxetine. In adolescents administered fluoxetine for 8 weeks, serum concentrations were significantly higher in females than males (111 vs. 62 ng/ml); similar sex differences were recorded after 12 weeks of treatment (197). Peak concentrations were 37% higher in adult women than men (198). Use of fluoxetine (as well as other SSRIs and TCAs) is commonly accompanied by increased hypothalamic-pituitary adrenal axis (HPA) activity (199). Serum cortisol concentrations were increased by 68% and 98% in depressed men and women respectively, after treatment with fluoxetine (200). Increased HPA axis activity may be related to treatment efficacy (201). Fluoxetine also induced significant increases in albumin concentrations in women only, and total serum tryptophan concentrations increased by 32% in men and 83% in women (200). Suicidal ideation was more likely to emerge in women than men during treatment (202).

Citalopram. Dose-corrected plasma concentrations in patients under 21 years of age were substantially higher in females than males (1.9 vs. 1.1 ng/ml) suggesting that sex differences in PKs manifest early in life (203). Dose-corrected serum concentrations were significantly higher in women than men ((204); 3.1 vs. 2.4 ng/ml). PET imaging indicated that following acute citalopram treatment women exhibited numerous areas of increased cortical glucose metabolism and relatively few areas of decreased cortical glucose metabolism, unlike men who exhibited many regions of decreased cortical glucose metabolism, with no regions of increased metabolism (205). Female sex was a risk factor for inappropriate secretion of antidiuretic hormone in patients on citalopram (206).

Sertraline. Plasma AUC was ~25% higher in young females than young males, and the terminal *t*_1/2_ elimination time was ~40% longer in young women; plasma concentrations were 35 to 40% lower in young men than in elderly or young women or elderly males (207–209). In a trial of olanzapine + sertraline vs. olanzapine + placebo for psychotic depression, women had significantly more delusions with disorganization than men, as well as higher cholesterol measures (210). The dropout rate for depressed patients treated with sertraline was 14% for women and 24% for men but this difference was not statistically significant; women were significantly more likely to report nausea and dizziness, and men were significantly more likely to report dyspepsia, sexual dysfunction, and increased urinary frequency (193).

Bupropion. Mean values for AUC, V_d_ normalized to body weight, C_max_ and elimination t_1/2_ were significantly greater in women than men; female:male ratios were ~1.3 for each measure (211). The odds of women having sharp EEG waves was significantly increased by a factor of 2.53 compared to men controlled for both age and dose. Similarly, women were 2.45 times more likely than men to have focal EEG slowing (212). Women have a 1.5-fold greater likelihood of having seizures than men (213).

Methylphenidate. The AUC was significantly greater in men than in women (93.4 vs. 73.5 ng·h/ml); women exhibited stronger subjective responses than men (214). Among teenagers with ADHD, once daily treatment resulted in more girls than boys exhibiting a comorbid anxiety disorder. Girls had a statistically superior response to methylphenidate at 1.5 h post-dosing and an inferior response at 12 h post-dosing (215). The authors argued that methylphenidate would be less likely to induce psychiatric ADRs (e.g., anxiety disorder) if dose titration were performed in patients based on systematic evidence of drug responses at different times across the day; no such dosing recommendations exist on the label (215).

Memantine. Women had about 45% higher exposure than men to memantine hydrochloride, but the difference in exposure was not present when body weight was taken into account (memantine label information). ADR ratio of 1.50, based on 8396 reports.

Desipramine. AUC and clearance rates were higher and lower, respectively, in women, but sample sizes were insufficiently powered to permit meaningful statistical evaluation (216). No sex differences were reported for desipramine clearance in children and adolescents (217). ADR ratio of 1.59, based on 2147 reports.

Clomipramine. Plasma concentrations were ~50% higher in women than men (191). ADR ratio of 1.57, based on 9072 reports.

Paroxetine. Women had 60% higher concentrations than men (28 vs. 16 ng/ml) although discrepant findings have been reported (218). Analysis of a clinical database of 218 paroxetine-treated panic disorder patients indicates that more men than women met the treatment response criteria (72 % vs. 55%; Steiner et al., 1996; cited by (219)). ADR ratio of 2.0, based on 63901 reports.

Fluvoxamine. In patients on monotherapy, plasma levels were approximately two-fold higher in women than men; curiously, the magnitude of the sex difference in plasma levels was greater at a lower dose (220). The biological basis of this sex effect has not been identified (208). The ADR ratio was 1.73, based on 8833 reports.

Mirtazapine. Women had significantly higher dose-corrected concentrations of mirtazapine and demethylmirtazapine and demethylmirtazapine:mirtazapine ratios than men (203) and Timmer et al. (221) documented 50% lower concentrations in men. ADR ratio of 1.53, based on 23241 reports.

*ATC Classification N7: Nervous system – Other nervous system drugs*

Tirilazad. For tirilazad mesylate, clearance values corrected for body weight were approximately 40% higher in young women than young men (222). In another report, mean tirilazad clearance after i.v. treatment was 60% higher in young women compared to middle-aged men, and clearance in middle-aged women was 31% higher than in age-matched men (223); the AUC was almost twice as high for men than women (223). Consistent with these PK values, a fixed dose design generated inappropriately low drug exposure in women (224). Consequently, in some countries tirilazad was approved for the treatment of subarachnoid hemorrhage in men but not women (225). No empirical reports of ADRs were identified in the literature, and too few case reports (n=7; 6 female) exist in *VigiBase*.

*ATC Classification P: Antiparasitics*

Primaquine. Women had approximately 2-fold higher median values for *C*_max_ (212 vs. 122 ng/ml) and AUC (1909 vs. 917 ng·h/ml) after treatment, which may put them at increased risk for toxicity when administered the same maintenance dose as men (121). In a study that enrolled fewer women than men, GI disturbances were reported more frequently in women (e.g., nausea 35% for females and 12% for males, (226)).

*ATC Classification R: Respiratory system*

Terfenadine. The C_max_ was significantly higher in women than men (227). Cardiac ADRs are also more common in women than men taking terfenadine; women treated with this antihistamine are more likely to exhibit TdP (228,229).

Fexofenadine. AUC was 33% higher in women than men, and C_max_ was 46% greater in females (230). Tracking performance and choice reaction time of women on driving tests was significantly worse than that of men (231).

Cetirizine. Clearance was slower in female than male children (0.59 vs. 0.77 l/h, (232)). The ADR ratio was 1.76 based on 25576 reports.

Mizolastine. The median duration of absorption of orally administered mizolastine was >3 h for men and only 40 min for women, contributing to striking variability in drug concentrations by sex (233). More than twice as many ADRs were reported for women than men in *VigiBase* (ratio of 2.23, based on 414 reports).

*Recreational drugs without WHO-ATC Classification Codes*

MDMA. Women cleared MDMA (methyl​enedioxy​methamphetamine) almost 40% slower than men (234). Women appear to be more sensitive to adverse side-effects of MDMA (Ecstacy) than men. MDMA significantly elevated plasma copeptin concentrations in women but not in men; an MDMA-induced increased production of the pre-pro-vasopressin precursor in women may account for hyponatremia typically reported in female Ecstasy users (235). Among heavy MDMA users, decreases in overall binding ratios in serotonergic neurons were detected in women but not men (236).

Cannabis (THC). Average plasma clearance rates are lower in women than men, 197 ±50 ml/min for women and 248 ±62 ml/min for men ((237) cited by (238). Cooper and Craft (2018) concluded that relative to men, women are more sensitive to both the therapeutic and adverse effects of CB1 receptor agonists such as THC in cannabis. Active cannabis significantly decreased pain sensitivity, indicative of analgesia, relative to inactive cannabis in men but not in women (239). Women exhibited signiﬁcantly greater subjective emotional responses to a 5 mg Δ^9^-THC dose than men, whereas men were more responsive to the 15 mg dose; however, this sex difference of pleasurable responses to THC may contribute to sex differences in the development of problematic cannabis use (240). When matched for cannabis use, women are more sensitive to the subjective effects of cannabis (~20% higher ratings of “good”, “liking” and “would take again”), which is related to greater cannabis abuse liability in women (241). Indeed, early onset cannabis use was associated with higher levels of introvertive anhedonia but only in female adolescents (242).

**References for Supplementary Data File**

1. Damholt B, Golor G, Wierich W, Pedersen P, Ekblom M, Zdravkovic M. An Open-Label, Parallel Group Study Investigating the Effects of Age and Gender on the Pharmacokinetics of the Once-Daily Glucagon-Like Peptide-1 Analogue Liraglutide. J Clin Pharmacol. 2006 Oct;46(6):635–41.

2. Flores Perez J, Juarez Olguin H, Flores Perez C, Perez Guille G, Guille Perez A, Camacho Vieyra A, et al. Effects of gender and phase of the menstrual cycle on the kinetics of ranitidine in healthy volunteers. Chronobiol Int. 2003 May;20(3):485–94.

3. Ehsanullah RS, Page MC, Tildesley G, Wood JR. Prevention of gastroduodenal damage induced by non-steroidal anti-inflammatory drugs: controlled trial of ranitidine. BMJ. 1988 Oct;297(6655):1017–21.

4. Loke YK, Singh S, Furberg CD. Long-term use of thiazolidinediones and fractures in type 2 diabetes: a meta-analysis. Can Med Assoc J. 2009 Oct;180(1):32–9.

5. Pritchard JF, Bryson JC, Kernodle AE, Benedetti TL, Powell JR. Age and gender effects on ondansetron pharmacokinetics: evaluation of healthy aged volunteers. Clin Pharmacol Ther. 1992 Jan;51(1):51–5.

6. Fadiran EO, Zhang L. Effects of Sex Differences in the Pharmacokinetics of Drugs and Their Impact on the Safety of Medicines in Women. In: Harrison-Woolrych M, editor. Medicines For Women. Cham: Springer International Publishing; 2015. p. 41–68.

7. Campbell NR, Hull RD, Brant R, Hogan DB, Pineo GF, Raskob GE. Different effects of heparin in males and females. Clin Invest Med. 1998 Apr;21(2):71–8.

8. Ndrepepa G, Schulz S, Neumann F-J, Byrne RA, Hoppmann P, Cassese S, et al. Bleeding after percutaneous coronary intervention in women and men matched for age, body mass index, and type of antithrombotic therapy. Am Heart J. 2013 Oct;166(3):534–40.

9. Gutiérrez-Chico JL, Mehilli J. Gender Differences in Cardiovascular Therapy: Focus on Antithrombotic Therapy and Percutaneous Coronary Intervention. Drugs. 2013 Oct;73(17):1921–33.

10. Ho P, Triggs E, Bourne D, Heazlewood V. The effects of age and sex on the disposition of acetylsalicylic acid and its metabolites. Br J Clin Pharmacol. 1985 Oct;19(5):675–84.

11. Coppe D, Wessinger SJ, Ransil BJ, Harris W, Salzman E. Sex differences in the platelet response to aspirin. Thromb Res. 1981 Oct;23(1–2):1–21.

12. Kelton JG, Carter CJ, Rosenfeld J, Massicotte-Nolan MP, Hirsh J. Sex-related differences in the efficacy of acetylsalicylic acid (ASA) : the absorption of ASA and its effect on collagen-induced thromboxane B2 generation. Thromb Res. 1981 Oct;24(1–2):163–8.

13. Buchanan MR, Rischke JA, Butt R, Turpie AG, Hirsh J, Rosenfeld J. The sex-related differences in aspirin pharmacokinetics in rabbits and man and its relationship to antiplatelet effects. Thromb Res. 1983 Jan;29(2):125–39.

14. Miners J, Grgurinovich N, Whitehead A, Robson R, Birkett D. Influence of gender and oral contraceptive steroids on the metabolism of salicylic acid and acetylsalicylic acid. Br J Clin Pharmacol. 1986 Oct;22(2):135–42.

15. Kjeldsen SE, Kolloch RE, Leonetti G, Malliond J-M, Zanchetti A, Elmfeldt D, et al. Influence of gender and age on preventing cardiovascular disease by antihypertensive treatment and acetylsalicylic acid. The HOT study. J Hypertens. 2000 Oct;18(5):629–42.

16. Yerman T, Gan WQ, Sin DD. The influence of gender on the effects of aspirin in preventing myocardial infarction. BMC Med. 2007 Oct;5(1):29.

17. Marcucci R, Cioni G, Giusti B, Fatini C, Rossi L, Pazzi M, et al. Gender and anti-thrombotic therapy: from biology to clinical implications. J Cardiovasc Transl Res. 2014 Feb;7(1):72–81.

18. Ridker PM, Cook NR, Lee I-M, Gordon D, Gaziano JM, Manson JE, et al. A randomized trial of low-dose aspirin in the primary prevention of cardiovascular disease in women. N Engl J Med. 2005 Mar;352(13):1293–304.

19. Koltai K, Papp J, Kenyeres P, Feher G, Tibold A, Alexy T, et al. Gender differences in hemorheological parameters and in in vitro platelet aggregation in acetylsalicylic acid and clopidogrel treated vascular patients. Biorheology. 2014;51(2–3):197–206.

20. Shen H, Herzog W, Drolet M, Pakyz R, Newcomer S, Sack P, et al. Aspirin Resistance in Healthy Drug-Naive Men Versus Women (from the Heredity and Phenotype Intervention Heart Study). Am J Cardiol. 2009 Oct;104(4):606–12.

21. Zuern CS, Lindemann S, Gawaz M. Platelet function and response to aspirin: gender-specific features and implications for female thrombotic risk and management. Semin Thromb Hemost. 2009 Apr;35(3):295–306.

22. Hobson AR, Qureshi Z, Banks P, Curzen N. Gender and Responses to Aspirin and Clopidogrel: Insights Using Short Thrombelastography. Cardiovasc Ther. 2009 Oct;27(4):246–52.

23. Ekström N, Cederholm J, Zethelius B, Eliasson B, Fhärm E, Rolandsson O, et al. Aspirin treatment and risk of first incident cardiovascular diseases in patients with type 2 diabetes: an observational study from the Swedish National Diabetes Register. BMJ Open. 2013 Oct;3(4):e002688.

24. Rydberg DM, Holm L, Mejyr S, Loikas D, Schenck-Gustafsson K, von Euler M, et al. Sex differences in spontaneous reports on adverse bleeding events of antithrombotic treatment. Eur J Clin Pharmacol. 2014 Oct;70(1):117–26.

25. Gong IY, Schwarz UI, Crown N, Dresser GK, Lazo-Langner A, Zou G, et al. Clinical and Genetic Determinants of Warfarin Pharmacokinetics and Pharmacodynamics during Treatment Initiation. PLoS One. 2011 Oct;6(11):e27808.

26. Takahashi H. Developmental changes in pharmacokinetics and pharmacodynamics of warfarin enantiomers in Japanese children. Clin Pharmacol Ther. 2000 Oct;68(5):541–55.

27. Whitley HP, Fermo JD, Chumney EC, Brzezinski WA. Effect of patient-specific factors on weekly warfarin dose. Ther Clin Risk Manag. 2007 Jun;3(3):499–504.

28. Sconce EA. The impact of CYP2C9 and VKORC1 genetic polymorphism and patient characteristics upon warfarin dose requirements: proposal for a new dosing regimen. Blood. 2005 Oct;106(7):2329–33.

29. Garcia D, Regan S, Crowther M, Hughes RA, Hylek EM. Warfarin Maintenance Dosing Patterns in Clinical Practice. Chest. 2005 Oct;127(6):2049–56.

30. Alotaibi GS, Almodaimegh H, McMurtry MS, Wu C. Do women bleed more than men when prescribed novel oral anticoagulants for venous thromboembolism? A sex-based meta-analysis. Thromb Res. 2013 Oct;132(2):185–9.

31. Humphries KH, Kerr CR, Connolly SJ, Klein G, Boone JA, Green M, et al. New-onset atrial fibrillation: sex differences in presentation, treatment, and outcome. Circulation. 2001 May;103(19):2365–70.

32. Jørgensen NR, Grove EL, Schwarz P, Vestergaard P. Clopidogrel and the risk of osteoporotic fractures: a nationwide cohort study: Clopidogrel and fractures. J Intern Med. 2012 Oct;272(4):385–93.

33. Soghomonyan S, Abdel-Rasoul M, Zuleta-Alarcon A, Grants I, Davila V, Yu J, et al. Clopidogrel IBS Patients Have Higher Incidence of Gastrointestinal Symptoms Influenced by Age and Gender. Dig Dis Sci. 2017 Oct;62(10):2728–43.

34. Stangier J. Clinical Pharmacokinetics and Pharmacodynamics of the Oral Direct Thrombin Inhibitor Dabigatran Etexilate: Clin Pharmacokinet. 2008 Oct;47(5):285–95.

35. Ciarambino T, Corbi G, Filippelli A, Regina M La, Para O, Tangianu F, et al. Anticoagulant drugs and gender: what is in the elderly? A minireview. J Gerontol Geriatr. 2019;123–6.

36. McConeghy KW, Bress A, Qato DM, Wing C, Nutescu EA. Evaluation of Dabigatran Bleeding Adverse Reaction Reports in the FDA Adverse Event Reporting System during the First Year of Approval. Pharmacother J Hum Pharmacol Drug Ther. 2014 Oct;34(6):561–9.

37. Mombelli G, Bosisio R, Calabresi L, Magni P, Pavanello C, Pazzucconi F, et al. Gender-related lipid and/or lipoprotein responses to statins in subjects in primary and secondary prevention. J Clin Lipidol. 2015 Oct;9(2):226–33.

38. Plakogiannis R, Arif SA. Women Versus Men: Is There Equal Benefit and Safety from Statins? Curr Atheroscler Rep. 2016 Oct;18(2):6.

39. Raparelli V, Pannitteri G, Todisco T, Toriello F, Napoleone L, Manfredini R, et al. Treatment and Response to Statins: Gender-related Differences. Curr Med Chem. 2017 Oct;24(24): 2628-38.

40. Kostis WJ, Cheng JQ, Dobrzynski JM, Cabrera J, Kostis JB. Meta-Analysis of Statin Effects in Women Versus Men. J Am Coll Cardiol. 2012 Oct;59(6):572–82.

41. Cangemi R, Romiti GF, Campolongo G, Ruscio E, Sciomer S, Gianfrilli D, et al. Gender related differences in treatment and response to statins in primary and secondary cardiovascular prevention: The never-ending debate. Pharmacol Res. 2017 Oct;117:148–55.

42. Makkar RR, Fromm BS, Steinman RT, Meissner MD, Lehmann MH. Female gender as a risk factor for torsades de pointes associated with cardiovascular drugs. JAMA. 1993 Dec;270(21):2590–7.

43. Werner D, Werner U, Meybaum A, Schmidt B, Umbreen S, Grosch A, et al. Determinants of Steady-State Torasemide Pharmacokinetics: Impact of Pharmacogenetic Factors, Gender and Angiotensin II Receptor Blockers. Clin Pharmacokinet. 2008 Oct;47(5):323–32.

44. Niemi M, Pasanen M, Neuvonen P. SLCO1B1 polymorphism and sex affect the pharmacokinetics of pravastatin but not fluvastatin. Clin Pharmacol Ther. 2006 Oct;80(4):356–66.

45. Ishikawa T, Mizuno K, Nakaya N, Ohashi Y, Tajima N, Kushiro T, et al. The Relationship Between the Effect of Pravastatin and Risk Factors for Coronary Heart Disease in Japanese Patients With Hypercholesterolemia. Circ J. 2008 Oct;72(10):1576–82.

46. Kang D, Verotta D, Schwartz J. Population analyses of amlodipine in patients living in the community and patients living in nursing homes. Clin Pharmacol Ther. 2006 Oct;79(1):114–24.

47. Beierle I, Meibohm B, Derendorf H. Gender differences in pharmacokinetics and pharmacodynamics. Int J Clin Pharmacol Ther. 1999 Nov;37(11):529–47.

48. Humphries KH, Izadnegahdar M, Sedlak T, Saw J, Johnston N, Schenck-Gustafsson K, et al. Sex differences in cardiovascular disease – Impact on care and outcomes. Front Neuroendocrinol. 2017 Oct;46:46–70.

49. Adams KF, Patterson JH, Gattis WA, O’Connor CM, Lee CR, Schwartz TA, et al. Relationship of Serum Digoxin Concentration to Mortality and Morbidity in Women in the Digitalis Investigation Group Trial. J Am Coll Cardiol. 2005 Oct;46(3):497–504.

50. Rathore SS, Wang Y, Krumholz HM. Sex-Based Differences in the Effect of Digoxin for the Treatment of Heart Failure. N Engl J Med. 2002 Oct;347(18):1403–11.

51. Harris RZ, Benet LZ, Schwartz JB. Gender Effects in Pharmacokinetics and Pharmacodynamics: Drugs. 1995 Oct;50(2):222–39.

52. Fife DJ, Maibach HI. Gender Differences in the Pharmacokinetics of Oral Dermatologic Medications. J Toxicol Cutan Ocul Toxicol. 2005 Oct;23(2):119–33.

53. Kang D. Population analyses of sustained-release verapamil in patients: Effects of sex, race, and smoking. Clin Pharmacol Ther. 2003 Oct;73(1):31–40.

54. Dadashzadeh S, Javadian B, Sadeghian S. The effect of gender on the pharmacokinetics of verapamil and norverapamil in human. Biopharm Drug Dispos. 2006 Oct;27(7):329–34.

55. Krecic-Shepard ME, Barnas CR, Slimko J, Jones MP, Schwartz JB. Gender-Specific Effects on Verapamil Pharmacokinetics and Pharmacodynamics in Humans. J Clin Pharmacol. 2000 Oct;40(3):219–30.

56. White W. Gender and age effects on the ambulatory blood pressure and heart rate responses to antihypertensive therapy. Am J Hypertens. 2001 Oct;14(12):1239–47.

57. Jarugula V, Yeh C-M, Howard D, Bush C, Keefe DL, Dole WP. Influence of Body Weight and Gender on the Pharmacokinetics, Pharmacodynamics, and Antihypertensive Efficacy of Aliskiren. J Clin Pharmacol. 2010 Oct;50(12):1358–66.

58. Israili Z. Clinical pharmacokinetics of angiotensin II (AT1) receptor blockers in hypertension. J Hum Hypertens. 2000 Oct;14(S1):S73–86.

59. Os I, Franco V, Kjeldsen SE, Manhem K, Devereux RB, Gerdts E, et al. Effects of Losartan in Women With Hypertension and Left Ventricular Hypertrophy: Results From the Losartan Intervention For Endpoint Reduction in Hypertension Study. Hypertension. 2008 Oct;51(4):1103–8.

60. Walle U, Fagan T, Topmiller M, Conradi E, Walle T. The influence of gender and sex steroid hormones on the plasma binding of propranolol enantiomers. Br J Clin Pharmacol. 1994 Oct;37(1):21–5.

61. Xie HG, Chen X. Sex differences in pharmacokinetics of oral propranolol in healthy Chinese volunteers. Zhongguo Yao Li Xue Bao. 1995 Sep;16(5):468–70.

62. MRC-Report. Report of Medical Research Council Working Party on mild to moderate hypertension: adverse reactions to bendrofluozide and propranolol for the treatment of mild hypertension. Lancet. 1981;2:539–43.

63. Roukoz H, Saliba W. Dofetilide: a new class III antiarrhythmic agent. Expert Rev Cardiovasc Ther. 2007 Oct;5(1):9–19.

64. Johnson JA, Akers WS, Herring VL, Wolfe MS, Sullivan JM. Gender Differences in Labetalol Kinetics: Importance of Determining Stereoisomer Kinetics for Racemic Drugs. Pharmacotherapy. 2000 Oct;20(6):622–8.

65. Krecic-Shepard M. Race and sex influence clearance of nifedipine: Results of a population study. Clin Pharmacol Ther. 2000 Oct;68(2):130–42.

66. Luzier A, Killian A, Wilton J, Wilson M, Forrest A, Kazierad D. Gender-related effects on metoprolol pharmacokinetics and pharmacodynamics in healthy volunteers. Clin Pharmacol Ther. 1999 Oct;66(6):594–601.

67. Sharma A, Pibarot P, Pilote S, Dumesnil JG, Arsenault M, Bélanger PM, et al. Toward Optimal Treatment in Women: The Effect of Sex on Metoprolol-Diphenhydramine Interaction. J Clin Pharmacol. 2010 Oct;50(2):214–25.

68. Ueno K, Sato H. Sex-related differences in pharmacokinetics and pharmacodynamics of anti-hypertensive drugs. Hypertens Res. 2012 Oct;35(3):245–50.

69. Wagg A, Franks B, Ramos B, Berner T. Persistence and adherence with the new beta-3 receptor agonist, mirabegron, versus antimuscarinics in overactive bladder: Early experience in Canada. Can Urol Assoc J. 2015 Oct;9(9–10):343.

70. Martan A, Masata J, Krhut J, Zachoval R, Hanus T, Svabik K. Persistence in the treatment of overactive bladder syndrome (OAB) with mirabegron in a multicenter clinical study. Eur J Obstet Gynecol Reprod Biol. 2017 Oct;210:247–50.

71. Wagg AS, Foley S, Peters J, Nazir J, Kool-Houweling L, Scrine L. Persistence and adherence with mirabegron vs antimuscarinics in overactive bladder: Retrospective analysis of a UK General Practice prescription database. Int J Clin Pract. 2017 Oct;71(10):e12996.

72. Kato D, Tabuchi H, Uno S. Safety, efficacy, and persistence of long-term mirabegron treatment for overactive bladder in the daily clinical setting: **i**nterim (1-year) report from a Japanese post-marketing surveillance study. LUTS Low Urin Tract Symptoms. 2019 Oct;11(1):14–23.

73. Kerbusch T, Wählby U, Milligan PA, Karlsson MO. Population pharmacokinetic modelling of darifenacin and its hydroxylated metabolite using pooled data, incorporating saturable first-pass metabolism, CYP2D6 genotype and formulation-dependent bioavailability: Population pharmacokinetics darifenacin. Br J Clin Pharmacol. 2003 Oct;56(6):639–52.

74. Hajdinjak T, Leskovar J. Darifenacin in a real-world practice: results of a 6-month phase IV trial. Zdr Vestnik-Slovenian Med J. 2013;82(5):288–97.

75. Rovner ES. Trospium Chloride in the Management of Overactive Bladder: Drugs. 2004 Oct;64(21):2433–46.

76. Liabeuf S, Gras V, Moragny J. Trospium chloride for overactive bladder may induce central nervous system adverse events. Eur Geriatr Med. 2014;5(3):220–4.

77. Magee MH, Blum RA, Lates CD, Jusko WJ. Prednisolone Pharmacokinetics and Pharmacodynamics in Relation to Sex and Race. J Clin Pharmacol. 2001 Oct;41(11):1180–94.

78. Bergmann TK, Barraclough KA, Lee KJ, Staatz CE. Clinical Pharmacokinetics and Pharmacodynamics of Prednisolone and Prednisone in Solid Organ Transplantation. Clin Pharmacokinet. 2012 Oct;51(11):711–41.

79. Lee I, Kaminski HJ, McPherson T, Feese M, Cutter G. Gender differences in prednisone adverse effects: Survey result from the MG registry. Neurol - Neuroimmunol Neuroinflammation. 2018 Oct;5(6):e507.

80. Lew KH, Ludwig EA, Milad MA, Donovan K, Jr. EM, Ferry JJ, et al. Gender-based effects on methylprednisolone pharmacokinetics and pharmacodynamics. Clin Pharmacol Ther. 1993 Oct;54(4):402–14.

81. Overholser BR, Kays MB, Forrest A, Sowinski KM. Sex-Related Differences in the Pharmacokinetics of Oral Ciprofloxacin. J Clin Pharmacol. 2004 Oct;44(9):1012–22.

82. Austin K, Mather L, Philpot C, McDonald P. Intersubject and dose-related variability after intravenous administration of erythromycin. Br J Clin Pharmacol. 1980 Oct;10(3):273–9.

83. Drici M-D, Knollmann BC, Wang W-X, Woosley RL. Cardiac Actions of Erythromycin: Influence of Female Sex: Surv Anesthesiol. 1999 Oct;43(3):181.

84. Colombo D, Banfi G, Cassano N, Graziottin A, Vena GA, et al. The GENDER ATTENTION Observational Study: Gender and Hormonal Status Differences in the Incidence of Adverse Events During Cyclosporine Treatment in Psoriatic Patients. Adv Ther. 2017 Oct;34(6):1349–63.

85. Mueller F, Büchel B, Köberle D, Schürch S, Pfister B, Krähenbühl S, et al. Gender-specific elimination of continuous-infusional 5-fluorouracil in patients with gastrointestinal malignancies: results from a prospective population pharmacokinetic study. Cancer Chemother Pharmacol. 2013 Oct;71(2):361–70.

86. Sloan JA, Loprinzi CL, Novotny PJ, Okuno S, Nair S, Barton DL. Sex Differences in Fluorouracil-Induced Stomatitis. J Clin Oncol. 2000 Oct;18(2):412.

87. Sloan JA, Goldberg RM, Sargent DJ, Vargas-Chanes D, Nair S, Cha SS, et al. Women experience greater toxicity with fluorouracil-based chemotherapy for colorectal cancer. J Clin Oncol. 2002 Mar;20(6):1491–8.

88. Chansky K, Benedetti J, Macdonald JS. Differences in toxicity between men and women treated with 5-fluorouracil therapy for colorectal carcinoma. Cancer. 2005 Oct;103(6):1165–71.

89. Joerger M. Quantitative Effect of Gender, Age, Liver Function, and Body Size on the Population Pharmacokinetics of Paclitaxel in Patients with Solid Tumors. Clin Cancer Res. 2006 Oct;12(7):2150–7.

90. Tuomainen PO, Ylitalo A, Niemelä M, Kervinen K, Pietilä M, Sia J, et al. Gender-Based Analysis of the 3-Year Outcome of Bioactive Stents Versus Paclitaxel-Eluting Stents in Patients with Acute Myocardial Infarction: An Insight from the TITAX-AMI Trial. J Invasive Cardiol. 2012;24(3):6.

91. Mikhail GW, Gerber RT, Cox DA, Ellis SG, Lasala JM, Ormiston JA, et al. Influence of Sex on Long-Term Outcomes After Percutaneous Coronary Intervention With the Paclitaxel-Eluting Coronary Stent. JACC Cardiovasc Interv. 2010 Oct;3(12):1250–9.

92. Cassidy J, Twelves C, Cameron D, Steward W, O’Byrne K, Jodrell D, et al. Bioequivalence of two tablet formulations of capecitabine and exploration of age, gender, body surface area, and creatinine clearance as factors influencing systemic exposure in cancer patients. Cancer Chemother Pharmacol. 1999;44(6):453–60.

93. Ilich AI, Danilak M, Kim CA, Mulder KE, Spratlin JL, Ghosh S, et al. Effects of gender on capecitabine toxicity in colorectal cancer. J Oncol Pharm Pract. 2016 Oct;22(3):454–60.

94. Fasanmade AA, Adedokun OJ, Ford J, Hernandez D, Johanns J, Hu C, et al. Population pharmacokinetic analysis of infliximab in patients with ulcerative colitis. Eur J Clin Pharmacol. 2009 Oct;65(12):1211–28.

95. Weisman MH, Moreland LW, Furst DE, Weinblatt ME, Keystone EC, Paulus HE, et al. Efficacy, pharmacokinetic, and safety assessment of adalimumab, a fully human anti-tumor necrosis factor-alpha monoclonal antibody, in adults with rheumatoid arthritis receiving concomitant methotrexate: A pilot study. Clin Ther. 2003 Oct;25(6):1700–21.

96. Zelinkova Z. Sex-dimorphic adverse drug reactions to immune suppressive agents in inflammatory bowel disease. World J Gastroenterol. 2012 Oct;18(47):6967.

97. Ochoa D, Prieto-Pérez R, Román M, Talegón M, Rivas A, Galicia I, et al. Effect of gender and *CYP2C9* and *CYP2C8* polymorphisms on the pharmacokinetics of ibuprofen enantiomers. Pharmacogenomics. 2015 Oct;16(9):939–48.

98. Knights K, McLean C, Tonkin A, Miners J. Lack of effect of gender and oral contraceptive steroids on the pharmacokinetics of (R)-ibuprofen in humans. Br J Clin Pharmacol. 1995 Oct;40(2):153–6.

99. Walker JS, Carmody JJ. Experimental Pain in Healthy Human Subjects: Gender Differences in Nociception and in Response to Ibuprofen: Surv Anesthesiol. 1999 Oct;43(4):239.

100. Butcher BE, Carmody JJ. Sex differences in analgesic response to ibuprofen are influenced by expectancy: A randomized, crossover, balanced placebo-designed study: Analgesic response to ibuprofen. Eur J Pain. 2012 Oct;16(7):1005–13.

101. Buchanan FF, Myles PS, Leslie K, Forbes A, Cicuttini F. Gender and Recovery After General Anesthesia Combined with Neuromuscular Blocking Drugs: Anesth Analg. 2006 Oct;102(1):291–7.

102. Niesters M, Dahan A, Kest B, Zacny J, Stijnen T, Aarts L, et al. Do sex differences exist in opioid analgesia? A systematic review and meta-analysis of human experimental and clinical studies: Pain. 2010 Oct;151(1):61–8.

103. Fillingim RB, Doleys DM, Edwards RR, Lowery D. Clinical Characteristics of Chronic Back Pain as a Function of Gender and Oral Opioid Use: Spine (Phila Pa 1976). 2003 Oct;28(2):143–50.

104. Rau R-H, Lin Y-C, Cheng J-K. Sex Differences in Elderly Patients Using Patient Controlled Analgesia in the Postoperative Period: A Retrospective Database Analysis. Int J Gerontol. 2016 Oct;10(3):146–50.

105. Viscusi ER, Gambling DR, Hughes TL, Manvelian GZ. Pharmacokinetics of extended-release epidural morphine sulfate: Pooled analysis of six clinical studies. Am J Heal Pharm. 2009 Oct;66(11):1020–30.

106. Sarton E, Kest B, Dahan A. Sex Differences in Morphine Analgesia. 2000;93(5):10.

107. Aubrun F, Salvi N, Coriat P, Riou B. Sex- and age-related differences in morphine requirements for postoperative pain relief. Anesthesiology. 2005 Jul;103(1):156–60.

108. Dahan A, Sarton E, Teppema L, Olievier C. Sex-related differences in the influence of morphine on ventilatory control in humans. Anesthesiology. 1998 Apr;88(4):903–13.

109. Fillingim RB, Ness TJ, Glover TL, Campbell CM, Hastie BA, Price DD, et al. Morphine responses and experimental pain: sex differences in side effects and cardiovascular responses but not analgesia. J Pain. 2005 Feb;6(2):116–24.

110. Riley JL, Hastie BA, Glover TL, Fillingim RB, Staud R, Campbell CM. Cognitive–Affective and Somatic Side Effects of Morphine and Pentazocine: Side-Effect Profiles in Healthy Adults. Pain Med. 2010 Oct;11(2):195–206.

111. Zacny JP. Morphine responses in humans: a retrospective analysis of sex differences. Drug Alcohol Depend. 2001 Oct;63(1):23–8.

112. Sadhasivam S, Chidambaran V, Olbrecht VA, Costandi A, Clay S, Prows CA, et al. Opioid-Related Adverse Effects in Children Undergoing Surgery: Unequal Burden on Younger Girls with Higher Doses of Opioids. Pain Med. 2015 Oct;16(5):985–97.

113. Lofwall MR, Nuzzo PA, Walsh SL. Effects of cold pressor pain on the abuse liability of intranasal oxycodone in male and female prescription opioid abusers. Drug Alcohol Depend. 2012 Oct;123(1–3):229–38.

114. Peniston JH, Xiang Q, Gould EM. Factors affecting acceptability of titrated oxymorphone extended release in chronic low back pain – an individual patient analysis. Curr Med Res Opin. 2010 Oct;26(8):1861–71.

115. McHugh RK, DeVito EE, Dodd D, Carroll KM, Potter JS, Greenfield SF, et al. Gender differences in a clinical trial for prescription opioid dependence. J Subst Abuse Treat. 2013 Oct;45(1):38–43.

116. Moody DE, Fang WB, Morrison J, McCance-Katz E. Gender differences in pharmacokinetics of maintenance dosed buprenorphine. Drug Alcohol Depend. 2011 Oct;118(2–3):479–83.

117. Garnaat SL, Weisberg RB, Uebelacker LA, Herman DS, Bailey GL, Anderson BJ, et al. The overlap of sleep disturbance and depression in primary care patients treated with buprenorphine. Subst Abus. 2017 Oct;38(4):450–4.

118. Tagarro I, Herrera J, Barutell C, D??ez MC, Mar??n M, Samper D, et al. Effect of a Simple Dose-Escalation Schedule on Tramadol Tolerability: Assessment in the Clinical Setting. Clin Drug Investig. 2005 Oct;25(1):23–31.

119. Seaber E, On N, Dixon RM, Gibbens M, Leavens WJ, Liptrot J, et al. The absolute bioavailability and metabolic disposition of the novel antimigraine compound zolmitriptan (311C90). Br J Clin Pharmacol. 1997 Oct;43(6):579–87.

120. Smith DA, Cleary EW, Watkins S, Huffman CS, Dilzer SC, Lasseter KC. Pharmacokinetics and Pharmacodynamics of Zolmitriptan in Patients with Mild to Moderate Hypertension: A Double-Blind, Placebo-Controlled Study. J Clin Pharmacol. 1998 Oct;38(8):685–93.

121. Quang NN, Chinh NT, Cuong BT, Binh VQ, Travers T, Dai B, et al. Sex Affects the Steady-State Pharmacokinetics of Primaquine but Not Doxycycline in Healthy Subjects. Am J Trop Med Hyg. 2009 Oct;81(5):747–53.

122. Sigtermans M, Dahan A, Mooren R, Bauer M, Kest B, Sarton E, et al. S(+)-ketamine Effect on Experimental Pain and Cardiac Output: A Population Pharmacokinetic-Pharmacodynamic Modeling Study in Healthy Volunteers. Anesthesiology. 2009 Oct;111(4):892–903.

123. Morgan CJA, Curran HV. Acute and chronic effects of ketamine upon human memory: a review. Psychopharmacology (Berl). 2006 Oct;188(4):408–24.

124. Routledge P, Stargel W, Kitchell B, Barchowsky A, Shand D. Sex-related differences in the plasma protein binding of lignocaine and diazepam. Br J Clin Pharmacol. 1981 Oct;11(3):245–50.

125. Nazir MS, Holdcroft A. Local anaesthetic drugs: adverse effects as reported through the ADROIT system in the UK. Pharmacoepidemiol Drug Saf. 2009 Oct;18(11):1000–6.

126. Loryan I, Lindqvist M, Johansson I, Hiratsuka M, van der Heiden I, van Schaik RHN, et al. Influence of sex on propofol metabolism, a pilot study: implications for propofol anesthesia. Eur J Clin Pharmacol. 2012 Oct;68(4):397–406.

127. Choong E, Loryan I, Lindqvist M, Nordling Å, el Bouazzaoui S, van Schaik RH, et al. Sex Difference in Formation of Propofol Metabolites: A Replication Study. Basic Clin Pharmacol Toxicol. 2013 Oct;113(2):126–31.

128. Hoymork SC, Raeder J. Why do women wake up faster than men from propofol anaesthesia? Br J Anaesth. 2005 Oct;95(5):627–33.

129. Li Y-Y, Ge D-J, Li J-Y, Qi B. Sex Differences in the Morphine-Sparing Effects of Intraoperative Dexmedetomidine in Patient-Controlled Analgesia Following General Anesthesia: A Consort-Prospective, Randomized, Controlled Clinical Trial. Medicine (Baltimore). 2016 Oct;95(18):e3619.

130. Davis GA, Rudy AC, Archer SM, Wermeling DP. Bioavailability of intranasal butorphanol administered from a single-dose sprayer. Am J Heal Pharm. 2005 Oct;62(1):48–53.

131. Gear RW, Miaskowski C, Gordon NC, Paul SM, Heller PH, Levine JD. Kappa-opioids produce significantly greater analgesia in women than in men. Nat Med. 1996 Nov;2(11):1248–50.

132. Abernethy DR, Divoll M, Greenblatt DJ, Ameer B. Obesity, sex, and acetaminophen disposition. Clin Pharmacol Ther. 1982 Jun;31(6):783–90.

133. Court MH, Zhu Z, Masse G, Duan SX, James LP, Harmatz JS, et al. Race, Gender, and Genetic Polymorphism Contribute to Variability in Acetaminophen Pharmacokinetics, Metabolism, and Protein-Adduct Concentrations in Healthy African-American and European-American Volunteers. J Pharmacol Exp Ther. 2017 Sep;362(3):431–40.

134. Sood S, Howell J, Sundararajan V, Angus PW, Gow PJ. Paracetamol overdose in Victoria remains a significant health-care burden. J Gastroenterol Hepatol. 2013 Aug;28(8):1356–60.

135. Li C, Martin BC. Trends in emergency department visits attributable to acetaminophen overdoses in the United States: 1993-2007. Pharmacoepidemiol Drug Saf. 2011 Oct;20(8):810–8.

136. Marino SE, Birnbaum AK, Leppik IE, Conway JM, Musib LC, Brundage RC, et al. Steady-State Carbamazepine Pharmacokinetics Following Oral and Stable-Labeled Intravenous Administration in Epilepsy Patients: Effects of Race and Sex. Clin Pharmacol Ther. 2012 Oct;91(3):483–8.

137. Besi E, Boniface DR, Cregg R, Zakrzewska JM. Comparison of tolerability and adverse symptoms in oxcarbazepine and carbamazepine in the treatment of trigeminal neuralgia and neuralgiform headaches using the Liverpool Adverse Events Profile (AEP). J Headache Pain. 2015 Oct;16(1):81.

138. Vickery PB, Tillery EE, DeFalco AP. Intravenous Carbamazepine for Adults With Seizures. Ann Pharmacother. 2018 Oct;52(3):285–9.

139. MacPhee GJ, Goldie C, Roulston D, Potter L, Agnew E, Laidlaw J, et al. Effect of carbamazepine on psychomotor performance in naive subjects. Eur J Clin Pharmacol. 1986;30(1):37–42.

140. Sudhop T, Bauer J, Elger CE, Bergmann K. Increased High-Density Lipoprotein Cholesterol in Patients with Epilepsy Treated with Carbamazepine: A Gender-Related Study. Epilepsia. 1999 Oct;40(4):480–4.

141. Boyd RA, Turck D, Abel RB, Sedman AJ, Bockbrader HN. Effects of Age and Gender on Single-Dose Pharmacokinetics of Gabapentin. Epilepsia. 1999 Oct;40(4):474–9.

142. Shaparin N, Slattum PW, Bucior I, Nalamachu S. Relationships Among Adverse Events, Disease Characteristics, and Demographics in Treatment of Postherpetic Neuralgia With Gastroretentive Gabapentin: Clin J Pain. 2015 Oct;31(11):983–91.

143. Vazquez B, Yang H, Williams B, Zhou S, Laurenza A. Perampanel efficacy and safety by gender: Subanalysis of phase III randomized clinical studies in subjects with partial seizures. Epilepsia. 2015 Oct;56(7):e90–4.

144. Wright CE, Sisson TL, Ichhpurani AK, Peters GR. Steady-State Pharmacokinetic Properties of Pramipexole in Healthy Volunteers. J Clin Pharmacol. 1997 Oct;37(6):520–5.

145. Hermann R. Effects of age and sex on the disposition of retigabine. Clin Pharmacol Ther. 2003 Oct;73(1):61–70.

146. Deeks ED. Retigabine (Ezogabine): In Partial-Onset Seizures in Adults with Epilepsy. CNS Drugs. 2011 Oct;25(10):887–900.

147. Melkersson KI, Hulting A-L, Rane AJ. Dose requirement and prolactin elevation of antipsychotics in male and female patients with schizophrenia or related psychoses: Gender differences and antipsychotics. Br J Clin Pharmacol. 2002 Oct;51(4):317–24.

148. Anderson SG, Livingston M, Couchman L, Smith DJ, Connolly M, Miller J, et al. Sex differences in plasma clozapine and norclozapine concentrations in clinical practice and in relation to body mass index and plasma glucose concentrations: a retrospective survey. Ann Gen Psychiatry. 2015 Oct;14(1):39.

149. Lane HY, Chang YC, Chang WH, Lin SK, Tseng YT, Jann MW. Effects of gender and age on plasma levels of clozapine and its metabolites: analyzed by critical statistics. J Clin Psychiatry. 1999 Jan;60(1):36–40.

150. Tang Y, Mao P, Li F-M, Li W, Chen Q, Jiang F, et al. Gender, age, smoking behaviour and plasma clozapine concentrations in 193 Chinese inpatients with schizophrenia. Br J Clin Pharmacol. 2007 Jul;64(1):49–56.

151. Ahmed M, Hussain I, O’Brien SM, Dineen B, Griffin D, McDonald C. Prevalence and associations of the metabolic syndrome among patients prescribed clozapine. Ir J Med Sci. 2008 Oct;177(3):205–10.

152. Ventriglio A, Baldessarini RJ, Vitrani G, Bonfitto I, Cecere AC, Rinaldi A, et al. Metabolic Syndrome in Psychotic Disorder Patients Treated With Oral and Long-Acting Injected Antipsychotics. Front Psychiatry. 2018;9:744.

153. West S, Rowbotham D, Xiong G, Kenedi C. Clozapine induced gastrointestinal hypomotility: A potentially life threatening adverse event. A review of the literature. Gen Hosp Psychiatry. 2017 Oct;46:32–7.

154. Nielsen J, Meyer JM. Risk factors for ileus in patients with schizophrenia. Schizophr Bull. 2012 May;38(3):592–8.

155. Lau SL, Muir C, Assur Y, Beach R, Tran B, Bartrop R, et al. Predicting Weight Gain in Patients Treated With Clozapine: The Role of Sex, Body Mass Index, and Smoking. J Clin Psychopharmacol. 2016 Apr;36(2):120–4.

156. Covell NH, Weissman EM, Essock SM. Weight gain with clozapine compared to first generation antipsychotic medications. Schizophr Bull. 2004;30(2):229–40.

157. Weiss U, Marksteiner J, Kemmler G, Saria A, Aichhorn W. Effects of Age and Sex on Olanzapine Plasma Concentrations. J Clin Psychopharmacol. 2005;25(6):5.

158. Aichhorn W, Marksteiner J, Walch T, Zernig G, Hinterhuber H, Stuppaeck C, et al. Age and Gender Effects on Olanzapine and Risperidone Plasma Concentrations in Children and Adolescents. J Child Adolesc Psychopharmacol. 2007 Oct;17(5):665–73.

159. Lipkovich I, Jacobson JG, Caldwell C, Hoffmann VP, Kryzhanovskaya L, Beasley CM. Early predictors of weight gain risk during treatment with olanzapine: analysis of pooled data from 58 clinical trials. Psychopharmacol Bull. 2009;42(4):23–39.

160. Castellani LN, Costa-Dookhan KA, McIntyre WB, Wright DC, Flowers SA, Hahn MK, et al. Preclinical and Clinical Sex Differences in Antipsychotic-Induced Metabolic Disturbances: A Narrative Review of Adiposity and Glucose Metabolism. J Psychiatry brain Sci. 2019;4.

161. Parekh A, Fadiran EO, Uhl K, Throckmorton DC. Adverse effects in women: implications for drug development and regulatory policies. Expert Rev Clin Pharmacol. 2011 Oct;4(4):453–66.

162. Eugene AR, Masiak J. A pharmacodynamic modelling and simulation study identifying gender differences of daily olanzapine dose and dopamine D2-receptor occupancy. Nord J Psychiatry. 2017 Oct;71(6):417–24.

163. Castberg I, Westin AA, Skogvoll E, Spigset O. Effects of age and gender on the serum levels of clozapine, olanzapine, risperidone, and quetiapine. Acta Psychiatr Scand. 2017 Oct;136(5):455–64.

164. Molden E, Waade RB, Hoff M, Haslemo T. Impact of Ageing on Serum Concentrations of Risperidone and Its Active Metabolite in Patients with Known *CYP2D6* Genotype. Basic Clin Pharmacol Toxicol. 2016 Oct;119(5):470–5.

165. Eberhard J, Lindstrom E, Holstad M, Levander S. Prolactin level during 5 years of risperidone treatment in patients with psychotic disorders. Acta Psychiatr Scand. 2007 Apr;115(4):268–76.

166. Yasui-Furukori N, Tsuchimine S, Saito M, Nakagami T, Sato Y, Kaneko S. Association between major Multidrug Resistance 1 (MDR1) gene polymorphisms and plasma concentration of prolactin during risperidone treatment in schizophrenic patients. Prog Neuro-Psychopharmacology Biol Psychiatry. 2007 Oct;31(6):1230–4.

167. Yasui-Furukori N, Saito M, Tsuchimine S, Nakagami T, Sato Y, Sugawara N, et al. Association between dopamine-related polymorphisms and plasma concentrations of prolactin during risperidone treatment in schizophrenic patients. Prog Neuro-Psychopharmacology Biol Psychiatry. 2008 Oct;32(6):1491–5.

168. Clevenger C V, Furth PA, Hankinson SE, Schuler LA. The Role of Prolactin in Mammary Carcinoma. Endocr Rev. 2003 Oct;24(1):1–27.

169. Usall J, Suarez D, Haro JM. Gender differences in response to antipsychotic treatment in outpatients with schizophrenia. Psychiatry Res. 2007 Dec;153(3):225–31.

170. Cabaleiro T, Ochoa D, López-Rodríguez R, Román M, Novalbos J, Ayuso C, et al. Effect of polymorphisms on the pharmacokinetics, pharmacodynamics, and safety of risperidone in healthy volunteers: risperidone pharmacogenetics. Hum Psychopharmacol Clin Exp. 2014 Oct;29(5):459–69.

171. Belmonte C, Ochoa D, Román M, Cabaleiro T, Talegón M, Sánchez-Rojas SD, et al. Evaluation of the Relationship Between Pharmacokinetics and the Safety of Aripiprazole and Its Cardiovascular Effects in Healthy Volunteers: J Clin Psychopharmacol. 2016 Oct;36(6):608–14.

172. Greenblatt DJ, Harmatz JS, Shader RI. Sex Differences in Diazepam Protein Binding in Patients with Renal Insufficiency. Pharmacology. 1978 Oct;16(1):26–9.

173. Greenblatt DJ, Allen MD, Harmatz JS, Shader RI. Diazepam disposition determinants. Clin Pharmacol Ther. 1980 Mar;27(3):301–12.

174. Macleod SM, Giles HG, Bengert B, Liu FF, Sellers EM. Age- and Gender-Related Differences in Diazepam Pharmacokinetics. J Clin Pharmacol. 1979 Oct;19(1):15–9.

175. Palva ES. Gender-related differences in diazepam effects on performance. Med Biol. 1985;63(2):92–5.

176. Farkas RH, Unger EF, Temple R. Zolpidem and Driving Impairment — Identifying Persons at Risk. N Engl J Med. 2013 Oct;369(8):689–91.

177. Greenblatt DJ, Harmatz JS, Singh NN, Steinberg F, Roth T, Moline ML, et al. Gender differences in pharmacokinetics and pharmacodynamics of zolpidem following sublingual administration: The Journal of Clinical Pharmacology. J Clin Pharmacol. 2014 Oct;54(3):282–90.

178. Verster JC, Roth T. Gender Differences in Highway Driving Performance After Administration of Sleep Medication: A Review of the Literature. Traffic Inj Prev. 2012 Oct;13(3):286–92.

179. Yang Y-H, Lai J-N, Lee C-H, Wang J-D, Chen P-C. Increased Risk of Hospitalization Related to Motor Vehicle Accidents Among People Taking Zolpidem: A Case–Crossover Study. J Epidemiol. 2011 Oct;21(1):37–43.

180. Norman JL, Fixen DR, Saseen JJ, Saba LM, Linnebur SA. Zolpidem prescribing practices before and after Food and Drug Administration required product labeling changes. SAGE Open Med. 2017 Oct;5:205031211770768.

181. Greenblatt DJ, Legangneux E, Harmatz JS, Weinling E, Freeman J, Rice K, et al. Dynamics and Kinetics of a Modified-Release Formulation of Zolpidem: Comparison With Immediate-Release Standard Zolpidem and Placebo. J Clin Pharmacol. 2006 Oct;46(12):1469–80.

182. Greenblatt D, Divoll M, Puri S, Ho I, Zinny M, Shader R. Clobazam kinetics in the elderly. Br J Clin Pharmacol. 1981;12(5):631–6.

183. Doty RL, Treem J, Tourbier I, Mirza N. A double-blind study of the influences of eszopiclone on dysgeusia and taste function. Pharmacol Biochem Behav. 2009 Oct;94(2):312–8.

184. Wong SL, Cao G, Mack RJ, Granneman GR. Pharmacokinetics of sertindole in healthy young and elderly male and female subjects*. Clin Pharmacol Ther. 1997 Oct;62(2):157–64.

185. Ereshefsky L, Saklad SR, Watanabe MD, Davis CM, Jann MW. Thiothixene pharmacokinetic interactions: a study of hepatic enzyme inducers, clearance inhibitors, and demographic variables. J Clin Psychopharmacol. 1991 Oct;11(5):296–301.

186. Greenblatt DJ, Divoll M, Harmatz JS, Shader RI. Oxazepam kinetics: effects of age and sex. J Pharmacol Exp Ther. 1980 Oct;215(1):86–91.

187. Divoll M, Greenblatt DJ, Harmatz JS, Shader RI. Effect of age and gender on disposition of temazepam. J Pharm Sci. 1981 Oct;70(10):1104–7.

188. Gaillot J, Heusse D, Hougton GW, Aurele M, Dreyfus JF. Pharmacokinetics and Metabolism of Zopiclone. Pharmacology. 1983 Oct;27(2):76–91.

189. Leufkens TRM, Vermeeren A. Zopiclone’s Residual Effects on Actual Driving Performance in a Standardized Test: A Pooled Analysis of Age and Sex Effects in 4 Placebo-Controlled Studies. Clin Ther. 2014 Oct;36(1):141–50.

190. Pratt LA. Antidepressant Use Among Persons Aged 12 and Over: United States, 2011–2014. 2017;(283):8.

191. Hildebrandt MG, Steyerberg EW, Stage KB, Passchier J, Kragh-Soerensen P, Group TDUA. Are Gender Differences Important for the Clinical Effects of Antidepressants? Am J Psychiatry. 2003 Oct;160(9):1643–50.

192. Vermeiden M, van den Broek W, Mulder P, Birkenhäger T. Influence of gender and menopausal status on antidepressant treatment response in depressed inpatients. J Psychopharmacol. 2010 Oct;24(4):497–502.

193. Kornstein SG, Schatzberg AF, Thase ME, Yonkers KA, McCullough JP, Keitner GI, et al. Gender Differences in Treatment Response to Sertraline Versus Imipramine in Chronic Depression. Am J Psychiatry. 2000 Oct;157(9):1445–52.

194. Baca E, Garcia-Garcia M, Porras-Chavarino A. Gender differences in treatment response to sertraline versus imipramine in patients with nonmelancholic depressive disorders. Prog Neuro-Psychopharmacology Biol Psychiatry. 2004 Oct;28(1):57–65.

195. Dahl ML, Bertilsson L, Nordin C. Steady-state plasma levels of nortriptyline and its 10-hydroxy metabolite: relationship to the CYP2D6 genotype. Psychopharmacology (Berl). 1996 Feb;123(4):315–9.

196. Pomara N, Shao B, Choi SJ, Tun H, Suckow RF. Sex-related differences in nortriptyline-induced side-effects among depressed patients. Prog Neuropsychopharmacol Biol Psychiatry. 2001 Jul;25(5):1035–48.

197. Blázquez A, Mas S, Plana MT, Gassó P, Méndez I, Torra M, et al. Plasma Fluoxetine Concentrations and Clinical Improvement in an Adolescent Sample Diagnosed With Major Depressive Disorder, Obsessive-Compulsive Disorder, or Generalized Anxiety Disorder: J Clin Psychopharmacol. 2014 Oct;34(3):318–26.

198. Ferguson JM, Hill H. Pharmacokinetics of Fluoxetine in Elderly Men and Women. Gerontology. 2006 Oct;52(1):45–50.

199. Manthey L, Leeds C, Giltay EJ, van Veen T, Vreeburg SA, Penninx BWJH, et al. Antidepressant use and salivary cortisol in depressive and anxiety disorders. Eur Neuropsychopharmacol. 2011 Oct;21(9):691–9.

200. Bano S, Akhter S, Afridi MI. Gender based response to fluoxetine hydrochloride medication in endogenous depression. J Coll Physicians Surg Pak. 2004 Mar;14(3):161–5.

201. Pariante CM. Risk Factors for Development of Depression and Psychosis: Glucocorticoid Receptors and Pituitary Implications for Treatment with Antidepressant and Glucocorticoids. Ann N Y Acad Sci. 2009 Oct;1179(1):144–52.

202. Perlis RH, Beasley Jr. CM, Wines Jr. JD, Tamura RN, Cusin C, Shear D, et al. Treatment-Associated Suicidal Ideation and Adverse Effects in an Open, Multicenter Trial of Fluoxetine for Major Depressive Episodes. Psychother Psychosom. 2007 Oct;76(1):40–6.

203. Reis M, Olsson G, Carlsson B, Lundmark J, Dahl M-L, Wålinder J, et al. Serum Levels of Citalopram and Its Main Metabolites in Adolescent Patients Treated in a Naturalistic Clinical Setting: J Clin Psychopharmacol. 2002 Oct;22(4):406–13.

204. Unterecker S, Riederer P, Proft F, Maloney J, Deckert J, Pfuhlmann B. Effects of gender and age on serum concentrations of antidepressants under naturalistic conditions. J Neural Transm. 2013 Oct;120(8):1237–46.

205. Munro CA, Workman CI, Kramer E, Hermann C, Ma Y, Dhawan V, et al. Serotonin modulation of cerebral glucose metabolism: Sex and age effects. Synapse. 2012 Oct;66(11):955–64.

206. Barclay TS, Lee AJ. Citalopram-associated SIADH. Ann Pharmacother. 2002 Oct;36(10):1558–63.

207. Ronfeld RA, Tremaine LM, Wilner KD. Pharmacokinetics of Sertraline and its N-Demethyl Metabolite in Elderly and Young Male and Female Volunteers: Clin Pharmacokinet. 1997 Oct;32(Supplement 1):22–30.

208. Preskorn SH. Clinically Relevant Pharmacology of Selective Serotonin Reuptake Inhibitors: An Overview with Emphasis on Pharmacokinetics and Effects on Oxidative Drug Metabolism. Clin Pharmacokinet. 1997 Oct;32(Supplement 1):1–21.

209. Warrington SJ. Clinical implications of the pharmacology of sertraline. Int Clin Psychopharmacol. 1991 Dec;6 Suppl 2:11–21.

210. Deligiannidis KM, Rothschild AJ, Barton BA, Kroll-Desrosiers AR, Meyers BS, Flint AJ, et al. A Gender Analysis of the Study of Pharmacotherapy of Psychotic Depression (STOP-PD): Gender and Age as Predictors of Response and Treatment-Associated Changes in Body Mass Index and Metabolic Measures. J Clin Psychiatry. 2013 Oct;74(10):1003–9.

211. Stewart JJ, Berkel HJ, Parish RC, Simar MR, Syed A, Bocchini JA, et al. Single-Dose Pharmacokinetics of Bupropion in Adolescents: Effects of Smoking Status and Gender. J Clin Pharmacol. 2001 Oct;41(7):770–8.

212. Macaluso M, Zackula R, D’Empaire I, Baker B, Liow K, Preskorn SH. Twenty Percent of a Representative Sample of Patients Taking Bupropion Have Abnormal, Asymptomatic Electroencephalographic Findings: J Clin Psychopharmacol. 2010 Oct;30(3):312–7.

213. Davidson J. Seizures and bupropion: a review. J Clin Psychiatry. 1989 Jul;50(7):256–61.

214. Patrick KS, Straughn AB, Minhinnett RR, Yeatts SD, Herrin AE, DeVane CL, et al. Influence of Ethanol and Gender on Methylphenidate Pharmacokinetics and Pharmacodynamics. Clin Pharmacol Ther. 2007 Oct;81(3):346–53.

215. Sonuga-Barke EJS, Coghill D, Markowitz JS, Swanson JM, Vandenberghe M, Hatch SJ. Sex Differences in the Response of Children With ADHD to Once-Daily Formulations of Methylphenidate. J Am Acad Child Adolesc Psychiatry. 2007 Oct;46(6):701–10.

216. Abernethy DR, Greenblatt DJ, Shader RI. Imipramine and desipramine disposition in the elderly. J Pharmacol Exp Ther. 1985 Jan;232(1):183–8.

217. Cohen LG, Biederman J, Wilens TE, Spencer TJ, Mick E, Faraone S V, et al. Desipramine Clearance in Children and Adolescents: Absence of Effect of Development and Gender. J Am Acad Child Adolesc Psychiatry. 1999 Oct;38(1):79–85.

218. Gex-Fabry M, Eap CB, Oneda B, Gervasoni N, Aubry J-M, Bondolfi G, et al. CYP2D6 and ABCB1 genetic variability: influence on paroxetine plasma level and therapeutic response. Ther Drug Monit. 2008 Aug;30(4):474–82.

219. Ballenger JC, Wheadon DE, Steiner M, Bushnell W, Gergel IP. Double-Blind, Fixed-Dose, Placebo-Controlled Study of Paroxetine in the Treatment of Panic Disorder. Am J Psychiatry. 1998 Oct;155(1):36–42.

220. Härtter S, Wetzel H, Hammes E, Hiemke C. Inhibition of antidepressant demethylation and hydroxylation by fluvoxamine in depressed patients. Psychopharmacology (Berl). 1993 Oct;110(3):302–8.

221. Timmer C, Paanakker J, Van Hal H. Pharmacokinetics of Mirtazapine from Orally Administered Tablets: Influence of Gender, Age and Treatment Regimen. Hum Psychopharmacol Clin Exp. 1996;11(6):497–509.

222. Hulst LK, Fleishaker JC, Peters GR, Harry JD, Wright DM, Ward P. Effect of age and gender on tirilazad pharmacokinetics in humans. Clin Pharmacol Ther. 1994 Apr;55(4):378–84.

223. Fleishaker JC, Hulst-Pearson LK, Peters GR. Effect of Gender and Menopausal Status on the Pharmacokinetics of Tirilazad Mesylate in Healthy Subjects. Am J Ther. 1995 Aug;2(8):553–60.

224. Marshall LF, Marshall SB. Pitfalls and Advances from the International Tirilazad Trial in Moderate and Severe Head Injury. J Neurotrauma. 1995 Oct;12(5):929–32.

225. Ann Panetta J, Srinivasan U. Gender Based Medicine. In: Annual Reports in Medicinal Chemistry. Elsevier; 1998. p. 355–63.

226. Nasveld P, Kitchener S, Edstein M, Rieckmann K. Comparison of tafenoquine (WR238605) and primaquine in the post-exposure (terminal) prophylaxis of vivax malaria in Australian Defence Force personnel. Trans R Soc Trop Med Hyg. 2002;96(6):683–4.

227. Honig PK, Wortham DC, Hull R, Zamani K, Smith JE, Cantilena LR. Itraconazole Affects Single-Dose Terfenadine Pharmacokinetics and Cardiac Repolarization Pharmacodynamics. J Clin Pharmacol. 1993 Oct;33(12):1201–6.

228. Ebert SN, Liu X-K, Woosley RL. Female Gender as a Risk Factor for Drug-Induced Cardiac Arrhythmias: Evaluation of Clinical and Experimental Evidence. J Women’s Heal. 1998 Oct;7(5):547–57.

229. US-GAO. Drug Safety: Most Drugs Withdrawn in Recent Years Had Greater Health Risks for Women [Internet]. 2001. p. 1–8. Available from: https://www.gao.gov/products/GAO-01-286R

230. Nicolas J-M, Espie P, Molimard M. Gender and interindividual variability in pharmacokinetics. Drug Metab Rev. 2009 Oct;41(3):408–21.

231. Vermeeren A, O’Hanlon JF. Fexofenadine’s effects, alone and with alcohol, on actual driving and psychomotor performance. J Allergy Clin Immunol. 1998 Oct;101(3):306–11.

232. Pitsiu M, Hussein Z, Majid O, Aarons L, De Longueville M, Stockis A. Retrospective population pharmacokinetic analysis of cetirizine in children aged 6 months to 12 years: Retrospective population pharmacokinetic analysis of cetirizine in children. Br J Clin Pharmacol. 2003 Oct;57(4):402–11.

233. Mesnil F, Mentre F, Dubruc C, Thenot J-P, Mallet A. Population Pharmacokinetic Analysis of Mizolastine and Validation from Sparse Data on Patients Using the Nonparametric Maximum Likelihood Method. J Pharmacokinet Biopharm. 1998;26(2):133–61.

234. Pardo-Lozano R, Farré M, Yubero-Lahoz S, O’Mathúna B, Torrens M, Mustata C, et al. Clinical Pharmacology of 3,4-Methylenedioxymethamphetamine (MDMA, “Ecstasy”): The Influence of Gender and Genetics (CYP2D6, COMT, 5-HTT). PLoS One. 2012 Oct;7(10):e47599.

235. Simmler LD, Hysek CM, Liechti ME. Sex Differences in the Effects of MDMA (Ecstasy) on Plasma Copeptin in Healthy Subjects. J Clin Endocrinol Metab. 2011 Oct;96(9):2844–50.

236. Reneman L, Booij J, de Bruin K, Reitsma JB, de Wolff FA, Gunning WB, et al. Effects of dose, sex, and long-term abstention from use on toxic effects of MDMA (ecstasy) on brain serotonin neurons. Lancet. 2001 Oct;358(9296):1864–9.

237. Wall ME, Sadler BM, Brine D, Taylor H, Perez-Reyes M. Metabolism, disposition, and kinetics of delta-9-tetrahydrocannabinol in men and women. Clin Pharmacol Ther. 1983 Sep;34(3):352–63.

238. Grotenhermen F. Pharmacokinetics and pharmacodynamics of cannabinoids. Clin Pharmacokinet. 2003;42(4):327–60.

239. Cooper ZD, Haney M. Sex-dependent effects of cannabis-induced analgesia. Drug Alcohol Depend. 2016 Oct;167:112–20.

240. Fogel JS, Kelly TH, Westgate PM, Lile JA. Sex differences in the subjective effects of oral Δ9-THC in cannabis users. Pharmacol Biochem Behav. 2017 Oct;152:44–51.

241. Cooper ZD, Haney M. Investigation of sex-dependent effects of cannabis in daily cannabis smokers. Drug Alcohol Depend. 2014 Oct;136:85–91.

242. Albertella L, Le Pelley ME, Copeland J. Cannabis use in early adolescence is associated with higher negative schizotypy in females. Eur Psychiatry. 2017 Oct;45:235–41.
